# Supplementary material for: Stochastic gene expression in proliferating cells: Differing noise intensity in single-cell and population perspectives
Source: PLoS Comput Biol. 2025 Jun 10;21(6):e1013014. doi: 10.1371/journal.pcbi.1013014 (PMC12151482; doi:10.1371/journal.pcbi.1013014)
Supplement: S1 Text — (PDF) [file pcbi.1013014.s001.pdf]

S1 Text. Supporting Information: *Stochastic Gene Expression in Proliferating Cells: Differing Noise Intensity in Single-Cell and Population Perspectives*

Zhanhao Zhang[a], Iryna Zabaikina[b,c], César Nieto[a], Zahra Vahdat[a,d,e], Pavol Bokes[b], and Abhyudai Singh[f,*]

[a]Department of Electrical and Computer Engineering, University of Delaware. Newark, DE, USA.
[b]Department of Applied Mathematics and Statistics, Comenius University, Bratislava Slovakia.
[c]Department of Mathematical Analysis and Numerical Mathematics, Comenius University, Bratislava, Slovakia.
[d]Dan L. Comprehensive Cancer Center, Baylor College of Medicine, Houston, Texas, United States of America.
[e]Department of Molecular and Cellular Biology, Baylor College of Medicine, Houston, Texas, United States of America.
[f]Department of Electrical and Computer Engineering, Biomedical Engineering, Mathematical Sciences, Center of Bioinformatics and Computational Biology, University of Delaware, Newark, Delaware, United States of America.
*Correspondence: absingh@udel.edu

1 Feedback in dilution. Single-cell model: Chapman–Kolmogorov equation

In the single-cell model we study the dynamics of protein concentration $x(t)$, which follows the following rules. Protein is produced instantly in portions (bursts) of random size. These bursts arrive at a rate λ and with a size drawn from an exponential distribution with mean β. The protein studied is considered to be relatively stable with a null degradation rate. Then, between the bursts, the protein dilutes exponentially with constant rate γ, which corresponds to the cell growth rate. Biologically, this means that the cell cycle duration is distributed following the exponential distribution with mean $1/\gamma$. In this appendix, we provide a solution approach for the protein pdf in the single cell model with the feedback in the protein dilution.

The time evolution of the probability density $p_{SC}(x,t)$ is described by the Chapman-Kolmogorov equation:

$$\frac{\partial p_{SC}(x,t)}{\partial t} = \frac{\partial}{\partial x}\left(x\frac{\gamma}{1+kx}p_{SC}(x,t)\right) + \frac{\lambda}{\beta}\int_0^x \mathrm{e}^{-(x-y)/\beta}p_{SC}(y,t)\,\mathrm{d}y - \lambda p_{SC}(x,t). \tag{1.1}$$

The first term of (1.1) captures the deterministic drift of probability due to dilution. The last two terms are related to stochastic dynamics. The integral represents the bursts that end at a concentration of x and the negative term is related to the jumps (bursts) when the concentration abandons the state x.

To find the stationary distribution, we write it as a probability conservation equation. It is done by collecting the last two terms of (1.1) as per Leibniz integral rule:

$$\frac{\lambda}{\beta}\int_0^x \mathrm{e}^{-(x-y)/\beta}p_{SC}(y,t)\,\mathrm{d}y - \lambda p_{SC}(x,t) = -\lambda\frac{\mathrm{d}}{\mathrm{d}x}\int_0^x \mathrm{e}^{-(x-y)/\beta}p(y)_{SC}\,\mathrm{d}y\,. \tag{1.2}$$

Then, we set $\partial p_{SC}/\partial t = 0$ in the expression (1.1), and integrate the result. It yields an integral equation:

$$\frac{x}{1+kx}p_{SC}(x) = \frac{\lambda}{\gamma}\int_0^x \mathrm{e}^{-(x-y)/\beta}p_{SC}(y)\,\mathrm{d}y\,. \tag{1.3}$$

The method to obtain the solution of (1.3) is based on the Laplace transform. We rearrange that equation in the following way:

$$xp_{SC}(x) = \frac{\lambda}{\gamma}\int_0^x (1+k(x-y)+ky)\mathrm{e}^{-(x-y)/\beta}p_{SC}(y)\,\mathrm{d}y\,. \tag{1.4}$$

It allows us to represent the right-hand side of the equation as the sum of three distinct convolutions:

$$xp_{SC}(x) = \frac{\lambda}{\gamma}\left((f*p_{SC})(x) + k(xf*p_{SC})(x) + k(f*xp_{SC})(x)\right), \tag{1.5}$$

where by f we denote the exponential function, that is, $f(x) = \mathrm{e}^{-x/\beta}$. By the asterisk in (1.5), we denote the convolution of two functions ($f*p_{SC} = \int_0^x p_{SC}(y)f(x-y)\,dy$), which helps us to solve the problem in the space of Laplace transforms.

As a quick introduction, we define the image $P(s) = \mathcal{L}\{p_{SC}\}(s)$, a function with argument s, as the Laplace transform of the function $p_{SC}(x)$ following the relationship:

$$P(s) = \mathcal{L}\{p_{SC}(x)\}(s) = \int_0^\infty p_{SC}(x)\mathrm{e}^{-sx}\,\mathrm{d}x\,, \tag{1.6}$$

with properties:

$$\mathcal{L}\{xp_{SC}(x)\}(s) = -\frac{\mathrm{d}P(s)}{\mathrm{d}s}, \tag{1.7a}$$

$$\mathcal{L}\{\mathrm{e}^{-x/\beta}*p_{SC}\}(s) = \frac{1}{s+\frac{1}{\beta}}P(s). \tag{1.7b}$$

Applying the Laplace transform to (1.5), it becomes a separable differential equation:

$$\frac{1}{P(s)}\frac{\mathrm{d}P(s)}{\mathrm{d}s} = \left(\frac{1}{s+1/\beta} - \frac{\lambda/\gamma+1}{s+1/\beta-\lambda k/\gamma}\right). \tag{1.8}$$

The general solution of (1.8) is given by:

$$P(s) = C\frac{s+1/\beta}{(s+1/\beta-\lambda k/\gamma)^{\lambda/\gamma+1}}, \tag{1.9}$$

where C is an arbitrary constant. Note that on the right-hand side there is a power function of the Laplace variable s, which is shifted by value $\eta = 1/\beta - \lambda k/\gamma$. In order to return to the original function $p_{SC}(x)$, we use the following relation [1]:

$$\mathcal{L}^{-1}\left\{(s+\eta)^{-a}\right\} = \frac{\mathrm{e}^{-\eta x}x^{a-1}}{\Gamma(a)}, \tag{1.10}$$

where $\mathcal{L}^{-1}$ is the inverse Laplace transform to (1.6). After applying it to (1.9), we obtain:

$$p_{SC}(x) = C\mathrm{e}^{-\eta x}x^{\lambda/\gamma-1}\frac{1+kx}{\Gamma(\lambda/\gamma)}.$$

We set C so that it satisfies the normalization condition $\int_0^\infty p_{SC}(x)dx = 1$ for the probability density function. This results in the stationary protein distribution:

$$p_{SC}(x) = \frac{\eta^2\beta}{\Gamma(\lambda/\gamma)}(\eta x)^{\lambda/\gamma-1}\mathrm{e}^{-\eta x}(1+kx), \quad \eta = 1/\beta - \lambda k/\gamma > 0. \tag{1.11}$$

The obtained density (1.11) is a mixture distribution, which can be represented as a weighted sum of two distinct gamma distributions:

$$p_{SC}(x) = (1-\lambda k\beta)f_{\text{Gamma}}\left(\frac{\lambda}{\gamma},\eta\right)(x) + \lambda k\beta f_{\text{Gamma}}\left(\frac{\lambda}{\gamma}+1,\eta\right)(x),$$

where by $f_{\text{Gamma}(\lambda/\gamma,\eta)}$ we denoted the probability density function of Gamma distribution with shape λ/γ and scale η. Note that this distribution is unimodal for any permissible values of parameters λ, β, γ, and k. This distribution has its peak at zero if $\lambda/\gamma < (4k\beta-1)/4(k\beta)^2$ when $k\beta > 1/4$, and if $\lambda/\gamma < 1$ when $k\beta < 1/4$; otherwise, the peak is non-zero.

By definition, the n-th raw moment is given by the integral $\langle x^n\rangle_{SC} = \int_0^\infty x^n p_{SC}(x)\,\mathrm{d}x$; we then use (1.11) to obtain a closed expression for the n-th raw moment:

$$\langle x^n\rangle_{SC} = (1+nk\beta)\frac{\Gamma\left(\frac{\lambda}{\gamma}+n\right)}{\eta^n\Gamma\left(\frac{\lambda}{\gamma}\right)}.$$

In the absence of the dilution regulation as $k = 0$, $p_{SC}(x)$ becomes the probability density function $p(x)$ of the unregulated gene expression [2]:

$$p(x) = \frac{1}{\beta\Gamma(\lambda/\gamma)}\left(\frac{x}{\beta}\right)^{\frac{\lambda}{\gamma}-1}\mathrm{e}^{-x/\beta}. \tag{1.12}$$

2 Feedback in dilution. Population model: Population balance equation

In this appendix, we study the feedback in the protein dilution, which is now implemented in the population model and affects not only the protein level but also the proliferation rate. Here, μ – the population growth rate – cannot be determined immediately due to the presence of feedback, which affects the cell cycle time and thus the population growth rate. An implicit assumption of the model is that cell volume is strictly increasing at rate $\gamma/(1+kx)$. Consequently, division events are governed by a nonhomogeneous Poisson process with rate $\gamma/(1+kx)$. An appropriate μ is required to keep the average cell volume constant, in order to prevent its infinite expansion or diminution to zero.

The expected population density $h(x,t)$ (number of cells with concentration x at time t) satisfies the population balance equation:

$$\frac{\partial h(x,t)}{\partial t} = \frac{\partial}{\partial x}\left(\frac{\gamma x}{1+kx}h(x,t)\right) + \frac{\gamma}{1+kx}h(x,t) + \lambda\int_0^x b(x-y)h(y,t)\,\mathrm{d}y - \lambda h(x,t), \tag{2.1}$$

where $b(x) = e^{-x/\beta}/\beta$ is pdf of the exponential distribution of the burst size.

We start by collapsing the last two terms according to the Leibniz integral rule as per (1.2). Subsequently, we use the Fourier method, that is, we assume that the population density function can be represented as a separable function:

$$h(x,t) = \mathrm{e}^{\mu t}p_{Pop}(x), \tag{2.2}$$

where the principal eigenvalue μ gives the population growth rate and the principal eigenvector $p_{Pop}(x)$ gives the protein distribution. Then (2.1) becomes

$$\left(\frac{1}{1+kx}-\frac{\mu}{\gamma}\right)p_{Pop}(x)+\frac{\mathrm{d}}{\mathrm{d}x}\left(\frac{xp_{Pop}(x)}{1+kx}\right)-\frac{\lambda}{\gamma}\frac{\mathrm{d}}{\mathrm{d}x}\left(\bar{\mathrm{B}}*p_{Pop}\right)(x)=0, \tag{2.3}$$

where $\bar{\mathrm{B}}(x)=e^{-x/\beta}$ is the complementary cumulative distribution function corresponding to $b(x)$.

We define an auxiliary function $q(x)=p_{Pop}(x)/(1+kx)$, which we substitute into the equation above; then we apply the Laplace transform as explained in Appendix 1, which gives us the relationship:

$$Q(s)-\frac{\mu}{\gamma}P(s)-sQ'(s)-\frac{\lambda}{\gamma}sB(s)P(s)=0, \tag{2.4}$$

where $P(s)$, $Q(s)$, and $B(s)$ are the Laplace images of functions $p_{Pop}(x)$, $q(x)$, and $\bar{\mathrm{B}}(x)$ respectively, defined as per (1.6). Applying the Laplace transform directly to the function $q(x)$, one can obtain:

$$P(s)=Q(s)-kQ'(s),$$

which is used to transform (2.4) into an ODE for $Q(s)$:

$$\frac{\mathrm{d}Q(s)}{Q(s)}=\frac{\frac{\mu}{\gamma}+\frac{\lambda}{\gamma}sB(s)-1}{\frac{\mu k}{\gamma}+\frac{\lambda k}{\gamma}sB(s)-s}\,\mathrm{d}s\,. \tag{2.5}$$

Despite the separable form of this equation, additional complexity is brought about by the generalization of the burst size distribution. However, since $\bar{\mathrm{B}}(x)$ corresponds to the exponential distribution, its Laplace image $B(s)$ is known:

$$B(s)=\int_0^\infty \bar{\mathrm{B}}(x)\mathrm{e}^{-sx}\,\mathrm{d}x=\frac{1}{s+1/\beta},\quad \mathrm{Re}\{s\}>-\frac{1}{\beta}. \tag{2.6}$$

We substitute (2.6) into (2.5) and obtain:

$$\frac{\mathrm{d}Q(s)}{Q(s)}=\frac{\frac{\gamma-\mu}{\gamma\beta}+s(1-\tilde{\mu})}{-\frac{\mu k}{\gamma\beta}-s\left(k\tilde{\mu}-\frac{1}{\beta}\right)+s^2}\,\mathrm{d}s\,,\quad \tilde{\mu}=\frac{\mu+\lambda}{\gamma}. \tag{2.7}$$

The right-hand side of (2.7) can be simplified by partial fraction decomposition. The quadratic in the denominator has two roots, which are real and given by

$$s_{1,2}=\frac{1}{2}\left(k\tilde{\mu}-\frac{1}{\beta}\pm\sqrt{D}\right),\qquad D=\left(k\tilde{\mu}+\frac{1}{\beta}\right)^2-\frac{4k}{\beta}\frac{\lambda}{\gamma}, \tag{2.8}$$

where $s_1>0$ and $s_2<0$ is for any positive values of λ,β,γ, and k. The partial fraction decomposition of (2.7) leads to:

$$\begin{aligned}\frac{\mathrm{d}Q(s)}{Q(s)}&=\frac{A_1}{s-s_1}+\frac{A_2}{s-s_2},\\ A_{1,2}&=\frac{1-\tilde{\mu}}{2}\pm\frac{1-\tilde{\mu}+2\lambda/\gamma+\beta k\tilde{\mu}(1-\tilde{\mu})}{2\beta\sqrt{D}}.\end{aligned} \tag{2.9}$$

The solution of (2.7) is

$$Q(s)=C(s-s_1)^{A_1}(s-s_2)^{A_2}. \tag{2.10}$$

The Laplace transform (2.10) must be analytic in the complex half-plane $\mathrm{Re}(s)>0$, implying that $A_1\in\{0,1,2,...\}$. In particular, the principal eigenvalue is obtained for $A_1=0$; it implies

$$\mu=\gamma-\frac{\lambda k\beta}{k\beta+1}. \tag{2.11}$$

Substituting (2.11) into (2.8) and (2.9), we obtain strictly negative values; we introduce additional variables for their opposite values:

$$\begin{aligned}s_2&=\frac{\lambda k}{\gamma(k\beta+1)}-\frac{1}{\beta},\qquad \rho=-s_2>0,\\ A_2&=-\frac{\lambda}{\gamma(k\beta+1)},\qquad \xi=-A_2>0.\end{aligned} \tag{2.12}$$

Inserting $s = 0$ into (2.4) and using the normalization condition $P(0) = 1$ yield $Q(0) = \mu$, which is used to find the value of C in (2.10). Finally, applying the inverse Laplace transform to (2.10) and returning to the initial function $p_{Pop}(x)$, we obtain:

$$p_{Pop}(x) = (1 + kx)\frac{\beta\rho^2}{\Gamma(\xi)}\mathrm{e}^{-\rho x}(\rho x)^{\xi-1}, \tag{2.13}$$

where the constants ρ and ξ are defined in (2.12).

As well as (1.11), stationary protein distribution $p_{Pop}(x)$ is a mixed distribution, i.e.,

$$p_{Pop}(x) = (1 - \xi k\beta)f_{\text{Gamma}}(\xi, \rho)(x) + \xi k\beta f_{\text{Gamma}}(\xi + 1, \rho)(x),$$

which is also unimodal for any set of parameters λ, β, γ, and k satisfying $\rho > 0$.

Note that without regulation, i.e. if $k = 0$, the pdf of the protein concentration in the population model is identical to the one in the single cell (1.12). Finally, we obtain explicit expressions for the n-th raw moment:

$$\langle x^n \rangle_{Pop} = (1 + nk\beta)\frac{\Gamma(\xi + n)}{\rho^n\Gamma(\xi)}.$$

3 Protein moments dynamics when single-cell protein distribution does not exists but population distribution exists

In this appendix, we demonstrate how the population distribution reaches the stationary distribution, but the single-cell distribution diverges. To illustrate this, we conducted simulations using parameters where the protein distribution at the single-cell level diverges but remains bounded from a population-level perspective. To achieve this, parameters satisfy the existence condition for (2.13) and do not satisfy one for (1.11). We set $\lambda = 1$ and choose such β, γ, k that $\gamma/k < \lambda\beta < \gamma/k + \gamma\beta$. The results of these simulations are presented in Fig A.

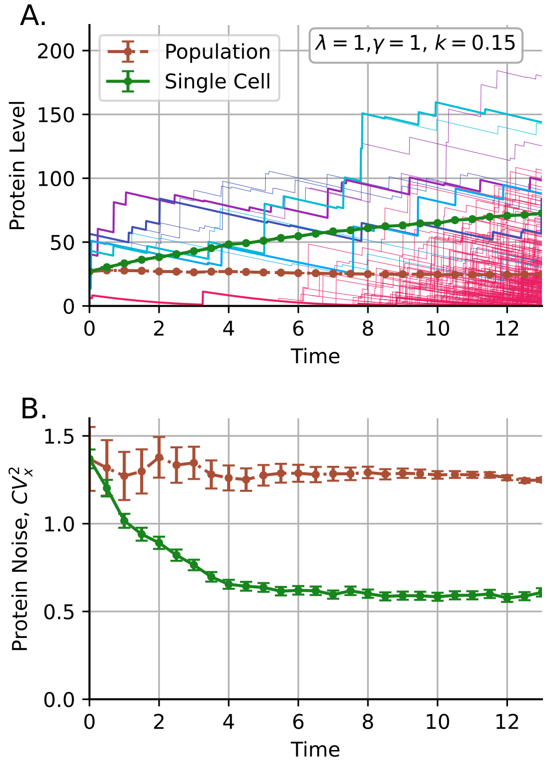

Fig A. Protein moments dynamics when single-cell protein distribution does not exist but population distribution exists. (**A**) Examples of protein trajectories. Each color in the background represents cells of the same colony. Green represents the statistics for a single cell approach while brown represent the population perspective. Error bars represents the 95% confidence interval. (**B**) Protein noise over time. Statistics were estimated as follows: for single cell 10 individuals were selected randomly with replacement from each colony, for population the statistics were calculated using all individuals of all colony replicas. 500 simulated colonies with an exponentially growing mean number. The average number of individuals per colony was of 180 after 13 units of time. The parameters values are $\lambda = 1$, $\gamma = 1$, $k = 0.15$.

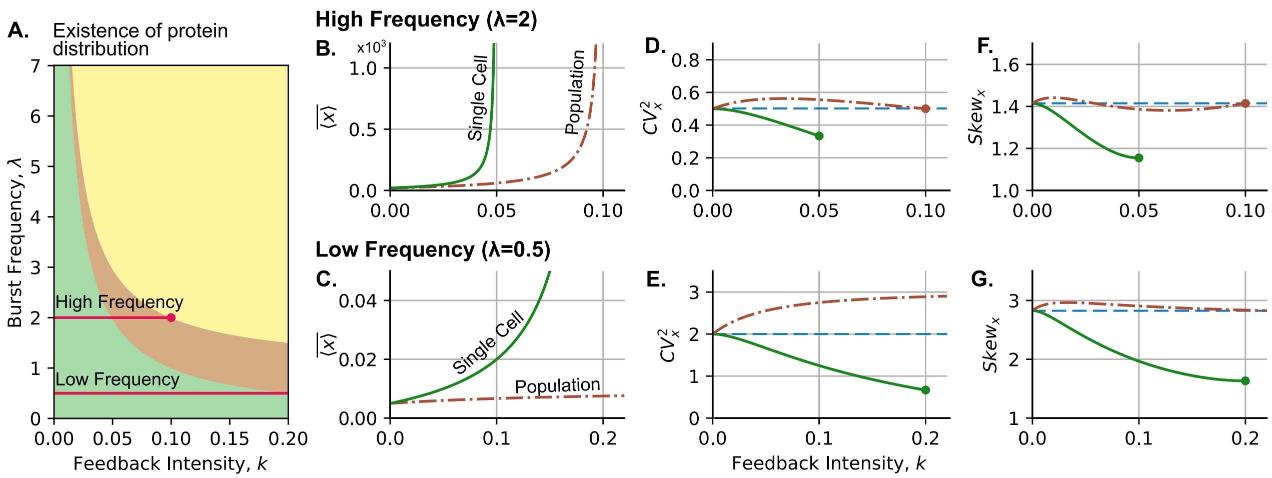

Fig B. Divergence of protein moments as the feedback intensity is increased keeping all other parameters fixed (**A**) Phase diagram of distribution existence similar to Fig 2A in the main text. Red line represents increase of k keeping $\lambda = 2$ and the low frequency $\lambda = 0.5$. Mean protein concentration: (**B**) for high frequency and (**C**) for low frequency. Protein noise: (**D**) for high frequency and (**E**) for low frequency. Protein Asymmetry: (**F**) for high frequency and (**G**) for low frequency. On (**B**)–(**G**) are shown statistics of single cell (green solid line) and population (brown dash-dotted line) compared to the unregulated case (blue horizontal dashed lines). Parameters: $\beta = 10$, $\gamma = 1$.

4 Moments analysis of the effects of the feedback strength while keeping the burst frequency constant

In the main text, we focus on the case when the burst frequency is adjusted so that the mean concentration remains fixed (7). In this appendix, we study protein noise and skewness as functions of feedback strength k, with parameters λ/γ and β being fixed.

In the single cell, the protein distribution $p_{SC}(x)$ (5) has the existence condition $\eta > 0$, from which it follows that k must be within the interval $K_{SC} = [0, 1/\overline{\langle x \rangle})$, with $\overline{\langle x \rangle} = \beta\lambda/\gamma$ for a given set of parameters (λ, β, γ). It is clear that as k approaches $1/\overline{\langle x \rangle}$, in the expression (6a), the mean value $\langle x \rangle_{SC}$ diverges due to a singularity in the denominator (Fig B(B), green line). To explore the behavior of the noise level (6b) and skewness (6c) on K_{SC}, we use the first derivative test. For the noise level, we find that $\partial(\mathrm{CV}_x^2)_{SC}/\partial k < 0$ meaning that $(\mathrm{CV}_x^2)_{SC}$ is a decreasing function of k within K_{SC} (Fig B(D), green line). We also use this approach for the skewness; its derivative is given by:

$$\frac{\partial(\mathrm{Skew}_x)_{SC}}{\partial k} = \frac{3\lambda\beta^2\eta(\beta\eta - 1)}{\gamma(1 + \lambda/\gamma - (\beta\eta)^2)^{5/2}}, \tag{4.1}$$

where the quadratic function in the denominator is always positive (it is concave and its two zeroes are not in K_{SC}), then $\partial(\mathrm{Skew}_x)_{SC}/\partial k < 0$ for any $k \in K_{SC}$. We find that both $(\mathrm{CV}_x^2)_{SC}$ and $(\mathrm{Skew}_x)_{SC}$ are monotonically decreasing functions with local maxima and minima being left and right endpoints of the interval K_{SC}, respectively. In particular:

$$\lim_{k \to 1/\overline{\langle x \rangle}} (\mathrm{CV}_x^2)_{SC} = \frac{\beta}{\overline{\langle x \rangle}} \frac{\lambda}{\lambda + \gamma},$$

$$\lim_{k \to 1/\overline{\langle x \rangle}} (\mathrm{Skew}_x)_{SC} = 2\frac{1}{\sqrt{\lambda/\gamma + 1}}.$$

In conclusion, for a given production flow $\lambda\beta$, the feedback of any strength reduces protein noise at the single cell level (green lines in Figs. BD–BE) and makes the distribution less skewed (green lines in Figs. BF–BG) compared to the unregulated expression (blue horizontal lines in corresponding figures).

We follow a similar approach for the statistics from the population perspective, where the permissible interval of k is $K_{Pop} = [0, 1/(\overline{\langle x \rangle} - \beta))$, for $\lambda > \gamma$ (the high frequency limit), and $K_{Pop} = [0, \infty)$, for $\lambda < \gamma$ (the low frequency limit). The behavior of mean, noise level, and skewness depends on the ratio λ/γ. In a low-frequency mode ($\lambda < \gamma$), we obtain:

$$\lim_{k \to \infty} \langle x \rangle_{Pop} = \frac{\overline{\langle x \rangle}}{1 - \lambda/\gamma}, \tag{4.2}$$

$$\lim_{k \to \infty} (\mathrm{CV}_x^2)_{Pop} = \frac{\beta}{\overline{\langle x \rangle}}(2 - \lambda/\gamma), \tag{4.3}$$

$$\lim_{k \to \infty} (\mathrm{Skew}_x)_{Pop} = 2\sqrt{\frac{\beta}{\overline{\langle x \rangle}}} \frac{(\lambda/\gamma)^2 - 3\lambda/\gamma + 3}{(2 - \lambda/\gamma)^{3/2}}. \tag{4.4}$$

Then the protein distribution in the population with the low transcriptional frequency ($\lambda < \gamma$) has higher, but always bounded statistics compared to the unregulated case. This behavior is shown in the second row of Fig B.

In high frequency mode ($\lambda > \gamma$) as k reaches the right endpoint of K_{Pop}, the mean value $\overline{\langle x \rangle}_{Pop}$ diverges and the statistics become identical to the unregulated case:

$$\lim_{k \to 1/(\overline{\langle x \rangle} - \beta)} (\mathrm{CV}_x^2)_{Pop} = \mathrm{CV}_x^2, \tag{4.5}$$

$$\lim_{k \to 1/(\overline{\langle x \rangle} - \beta)} (\mathrm{Skew}_x)_{Pop} = \mathrm{Skew}_x, \tag{4.6}$$

which is shown in the first row of Fig B.

The first derivative of the squared coefficient of variation,

$$\frac{\partial (\mathrm{CV}_x^2)_{Pop}}{\partial k} = \frac{\beta}{\overline{\langle x \rangle}} \frac{(1 - 2\lambda)k\beta + 1}{1 + k\beta},$$

has a single root at $1/(2\lambda - 1)\beta$, indicating that over the interval K_{Pop}, $(\mathrm{CV}_x^2)_{Pop}$ is monotonically increasing if $2\lambda < \gamma$; otherwise, it is concave (Figs. BD–BE). The first derivative test for skewness involves analysis of a cubic equation, which was done numerically. We conclude that the low frequency leads to $(\mathrm{Skew}_x)_{Pop} > \mathrm{Skew}_x$ on the whole interval K_{Pop}, maximum is reached within K_{SC} (Fig B(G)). The high frequency leads to minor fluctuations of $(\mathrm{Skew}_x)_{Pop}$ around Skew_x with single intersection within K_{Pop} (Fig B(F)).

Overall, we use the statistics of the unregulated case as a critical point for comparison of both perspectives. We conclude that for given production rate $\lambda\beta$ and admissible values of $k > 0$ protein distribution in the population is always noisier and more right-skewed compared to the single-cell one.

5 Protein distribution for a transcription rate proportional to the cell growth rate

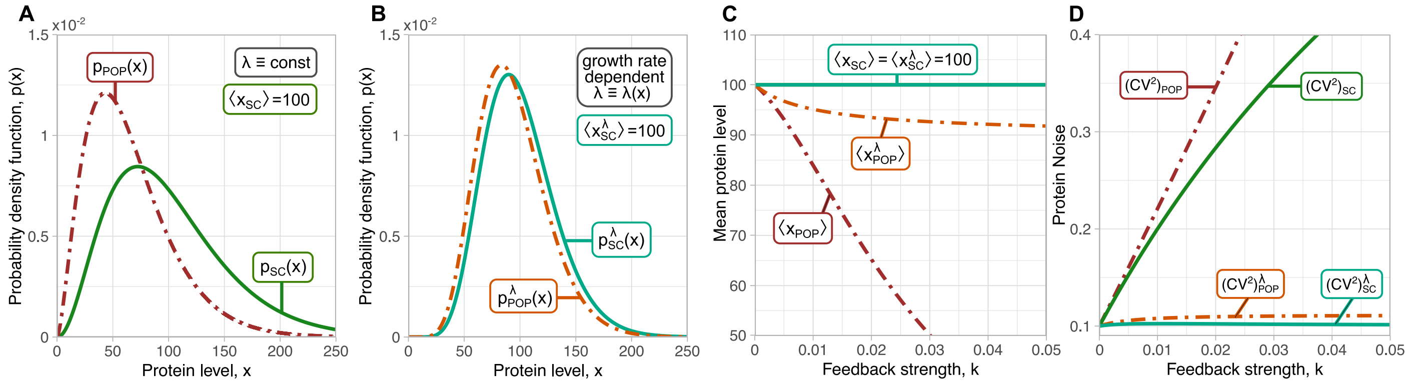

Fig C. Difference between single cell and population perspectives is less prominent when the burst frequency is proportional to the cell growth rate. Comparison of protein distributions in single-cell and population perspectives as: (**A**) the burst frequency is independent from protein concentration, λ_{max} is chosen so that $\overline{\langle x \rangle}_{SC} = 100$ (case studied in our article); (**B**) the burst frequency is protein-dependent, λ_{max} is again chosen so that $\overline{\langle x \rangle}^{\lambda}_{SC} = 100$ as per (5.3). (**C**) Mean protein concentration and (**D**) protein noise in both models. For (**A**) and (**B**), we set $k = 0.02$. For all plots, we set $\beta = 10$ and $\gamma = 1$.

In this appendix, we study the effect of a transcription rate proportional to the growth rate. We modify the model presented in the main text, so that the burst events occur with a frequency proportional to the protein-dependent growth rate. Compared to (1), this can be represented by jumps in the gene product x as:

$$x \xrightarrow{\lambda(x)} x + b, \tag{5.1}$$

in which $\lambda(x)$, the burst frequency, is proportional to the dilution/growth rate, i.e.,

$$\lambda(x) = \frac{\lambda_{max}}{1 + kx} = \frac{\tilde{\lambda}\gamma}{1 + kx} = \tilde{\lambda}\gamma(x), \tag{5.2}$$

where $\tilde{\lambda}$ is the maximum burst frequency λ_{max} normalized to the maximum dilution rate γ. We formulate and solve the dCKE following the approach provided in Appendix 1. In this case, the steady-state protein distribution in the single-cell model is as follows:

$$p^{\lambda}_{SC}(x) = \frac{1 + kx}{\beta\Gamma(\tilde{\lambda})(1 + \tilde{\lambda}k\beta)} e^{-\frac{x}{\beta}} \left(\frac{x}{\beta}\right)^{\tilde{\lambda}-1}.$$

Here and below we use superscript λ to mark distributions and moments for the model with the protein-dependent burst frequency.

The n-th steady-state raw moment of the protein concentration in the single cell follows:

$$\overline{\langle x^n \rangle}^{\lambda}_{SC} = \beta^n (\tilde{\lambda})_n \frac{1 + k\beta(\tilde{\lambda} + n)}{1 + \tilde{\lambda} k \beta},$$

where $(\tilde{\lambda})_n$ is the Pochhammer symbol, defined as $(\tilde{\lambda})_n = \Gamma(\tilde{\lambda} + n)/\Gamma(\tilde{\lambda})$, with $\Gamma(\cdot)$ being the gamma function. The mean protein level (given that $n = 1$) follows:

$$\overline{\langle x \rangle}^{\lambda}_{SC} = \tilde{\lambda}\beta \frac{1 + k\beta(\tilde{\lambda} + 1)}{1 + \tilde{\lambda} k \beta}. \tag{5.3}$$

Then we proceed and solve the population balance equation (see Appendix 2). The steady-state protein distribution for the population perspective results in:

$$p^{\lambda}_{POP}(x) = \frac{1 + kx}{\beta \Gamma(u)(1 + uk\beta)} e^{-\frac{x}{\beta}} \left(\frac{x}{\beta}\right)^{u-1}, \quad u = \frac{\tilde{\lambda}}{1 + \tilde{\mu} k \beta},$$

where $\tilde{\mu}$ is the population growth rate normalized to γ and is given by:

$$\tilde{\mu} = \frac{-(1 + k\beta(\tilde{\lambda} - 1)) + \sqrt{(1 + k\beta(\tilde{\lambda} - 1))^2 + 4k\beta}}{2k\beta}.$$

The n-th steady-state raw moment of the protein concentration in the cell population is following:

$$\overline{\langle x^n \rangle}^{\lambda}_{POP} = \beta^n (u)_n \frac{1 + k\beta(u + n)}{1 + uk\beta}.$$

In Fig C, we illustrate the effect of increasing the feedback intensity when the burst frequency is coupled with the dilution/proliferation rate according to (5.2). Fig C(A) provides a reference by showing the difference in distributions between both perspectives when the burst frequency is independent of the dilution rate, for a feedback strength of $k = 0.02$. In Fig C(B), using the same feedback strength ($k = 0.02$), we use (5.3) to chose $\lambda_{\max}$ so that the mean protein level at the single-cell level is set to $\overline{\langle x \rangle}^{\lambda}_{SC} = 100$. The resultant distribution from a population perspective is also shown with these parameters.

Fig C(C) shows how the mean protein levels diverge increasing the feedback strength. To obtain this graph, we set $\lambda_{\max}$ for each model (coupling and not coupling) so that the mean protein level in the single-cell perspective is set to $\langle x \rangle_{SC} = 100$ using (5.3). The mean for the population perspective is estimated using the respective $\lambda_{\max}$. Similarly, Fig C(D) plots the noise measured using the squared coefficient of variability. This figure demonstrates that coupling the burst frequency and growth rate results in fewer differences between single-cell and population approaches. In the limit of strong feedback, the gap between the metrics in both population perspectives approximately reaches a steady value.

6 Protein distribution when the protein is short-lived

In the main article, we assumed that the protein half-life is much longer than the cell-cycle average time. If we relax the assumption of no degradation and instead assume that the protein has a half-life comparable with the cell cycle duration, it will spontaneously degrade at a constant rate $\gamma_{\text{deg}} > 0$ (here and below, we add "deg" to the article notation to distinguish the new model with non-zero degradation rate). This affects only the deterministic decay of the protein between consecutive bursts, now described by:

$$\dot{x}(t) = -\frac{\gamma x}{1 + kx} - \gamma_{\text{deg}} x.$$

Following the same solution approach as in Appendix 1, we obtain that in a single-cell perspective, the steady-state distribution is the following:

$$p^{\text{deg}}_{SC}(x) = C\psi(1 + kx)(\psi x)^{\frac{\lambda}{\gamma + \gamma_{\text{deg}}} - 1} (\psi x + 1)^{\frac{\lambda\gamma}{\gamma_{\text{deg}}(\gamma + \gamma_{\text{deg}})} - 1} e^{-x/\beta}, \tag{6.1}$$

where $\psi = \gamma_{\text{deg}} k/(\gamma + \gamma_{\text{deg}})$ is a positive constant and

$$C = \left(U\left(\frac{\lambda\psi}{\gamma_{\text{deg}} k}, \frac{\lambda}{\gamma_{\text{deg}}}, \frac{1}{\psi\beta}\right) + \frac{\lambda}{\gamma_{\text{deg}}} U\left(\frac{\lambda\psi}{\gamma_{\text{deg}} k} + 1, \frac{\lambda}{\gamma_{\text{deg}}} + 1, \frac{1}{\psi\beta}\right) \right)^{-1}.$$

By $U(\cdot, \cdot; \cdot)$ we denote the Tricomi function (the confluent hypergeometric function of the second kind).

The protein distribution (6.1) yields the n-th steady-state raw moment of the protein concentration:

$$\begin{aligned} \overline{\langle x^n \rangle}^{\text{deg}}_{SC} = C\psi^{-n} \left(\frac{\lambda\psi}{\gamma_{\text{deg}} k}\right)_n \cdot \Bigg(& U\left(\frac{\lambda\psi}{\gamma_{\text{deg}} k} + n, \frac{\lambda}{\gamma_{\text{deg}}} + n, \frac{1}{\psi\beta}\right) \\ & + \left(\frac{\lambda}{\gamma_{\text{deg}}} + \frac{nk}{\psi}\right) U\left(\frac{\lambda\psi}{\gamma_{\text{deg}} k} + n + 1, \frac{\lambda}{\gamma_{\text{deg}}} + n + 1, \frac{1}{\psi\beta}\right) \Bigg). \end{aligned} \tag{6.2}$$

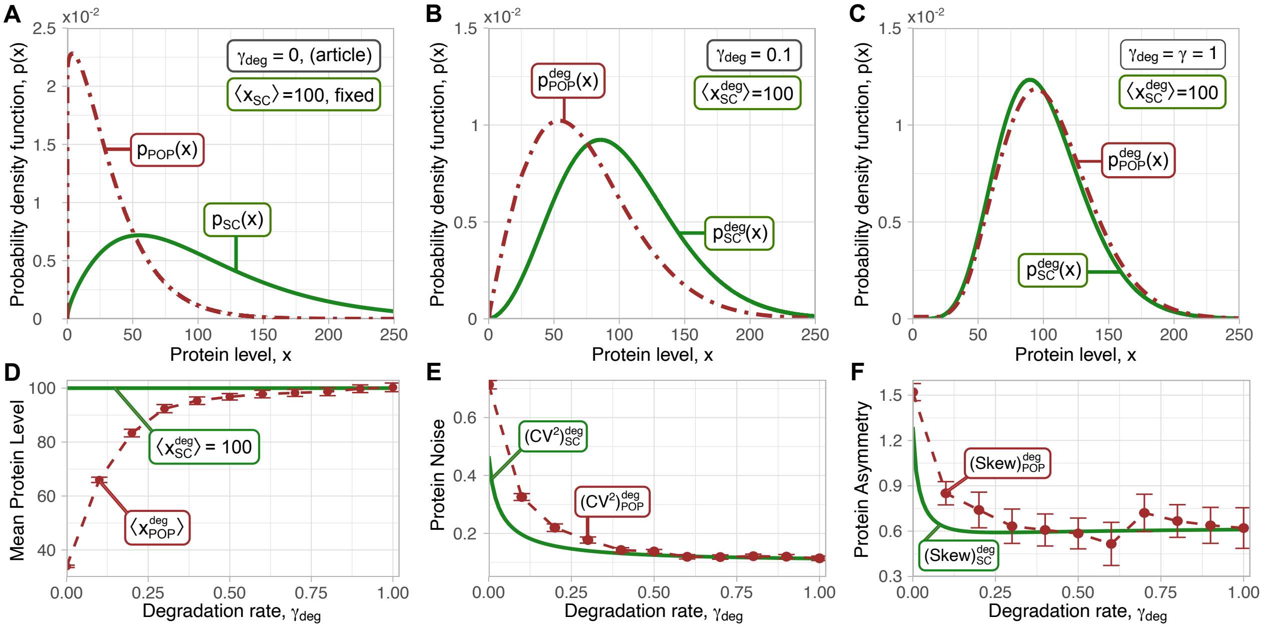

Fig D. When the gene product is unstable, the distributions for single cell and population converge. Comparison of protein distributions in single-cell (green solid lines) and population (red dot-dashed lines) perspectives as: (**A**) the protein is stable and the degradation rate is equal to zero (case studied in the article); **(B)** the degradation rate is slower then the maximum dilution rate ($\gamma_{\text{deg}} = \gamma/10 = 0.1$); **(C)** the degradation rate is equal to the maximum dilution rate ($\gamma_{\text{deg}} = \gamma = 1$). **(D)** Mean protein concentration, **(E)** Protein noise quantified by the squared coefficient of variation and **(E)** The protein asymmetry quantified by the skewness for both frameworks: single cell (green) and population (brown error bars). In all cases, the burst frequency λ is set such that $\overline{\langle x\rangle}_{SC} = \overline{\langle x\rangle}^{\text{deg}}_{SC} = 100$ using (6.3). Other parameters: $k = 0.05$, $\beta = 10$, $\gamma = 1$. The moments for the population perspective were calculated from 2000 simulated colonies. The error bars represent 95% confidence intervals using bootstrapping. The distributions $p^{\text{deg}}_{SC}(x)$ are given by (6.1) and $p^{\text{deg}}_{POP}(x)$ is obtained using boundary-corrected density estimation from simulations.

The mean protein concentration in a single cell is (Fig D(B)):

$$\overline{\langle x\rangle}^{\text{deg}}_{SC} = \frac{C\lambda}{\gamma_{\text{deg}}k}\left(U\left(\frac{\lambda\psi}{\gamma_{\text{deg}}k}+1, \frac{\lambda}{\gamma_{\text{deg}}}+1, \frac{1}{\psi\beta}\right)+\frac{\lambda+\gamma+\gamma_{\text{deg}}}{\gamma_{\text{deg}}}U\left(\frac{\lambda\psi}{\gamma_{\text{deg}}k}+2, \frac{\lambda}{\gamma_{\text{deg}}}+2, \frac{1}{\psi\beta}\right)\right). \tag{6.3}$$

The analytical results (6.1), (6.2), and (6.3) were validated through simulations of the corresponding model.

Including natural degradation in the population model significantly increases complexity. Specifically, the ODE for the probability distribution $p^{\text{deg}}_{POP}(x)$ becomes a confluent form of the Heun equation. Solving this equation remains an area for future research; hence, we relied on simulations to demonstrate the effects of γ_{deg} on the population distribution.

Compared to long-lived proteins, the steady-state distribution for short-lived proteins exists for any set of positive parameters $\alpha, \beta, k, \gamma, \gamma_{\text{deg}}$. This result is expected, as the existence conditions were initially caused by the accumulation of protein in a cell due to the cell-growth-dependent dilution rate. Adding an independent source of protein concentration decrease – natural degradation – resolves this issue. The simulations showed that $p^{\text{deg}}_{POP}(x)$ also exists for any set of positive parameters $\alpha, \beta, k, \gamma, \gamma_{\text{deg}}$, following the same reasoning as in the single-cell case.

We compare the obtained results with the single-cell and population models of long-lived protein (i.e. case studied in the main text), which is shown in Fig D(A). For correct comparison, we hold the mean protein level in all single-cell models fixed to the same value ($\overline{\langle x\rangle}^{\text{deg}}_{SC} = \overline{\langle x\rangle}_{SC} = 100$). When the dilution rate is the dominant cause of protein decay (i.e., $\gamma > \gamma_{\text{deg}}$), $p^{\text{deg}}_{SC}(x)$ and $p^{\text{deg}}_{POP}(x)$ are close to $p_{SC}(x)$ and $p_{POP}(x)$, respectively (Fig D(B)). As degradation becomes dominant ($\gamma \leq \gamma_{\text{deg}}$), the feedback effect becomes more negligible, leading to almost unregulated protein decay. Thus both distributions $p^{\text{deg}}_{SC}(x)$ and $p^{\text{deg}}_{POP}(x)$ become almost identical (Fig D(C)). As γ_{deg} increases further, both distributions approach the gamma distribution corresponding to the unregulated case ($p_{\text{unreg}}(x) = f_{Gamma}(\lambda/\gamma_{\text{deg}}, \beta)$). The statistics (Fig D(D–F)) confirm this observation. While the degradation rate remains low, there is a significant difference between the single-cell and population-level statistics. However, these statistics rapidly converge to those of the gamma distribution described earlier, aligning with (4). It is noteworthy that this transition in behavior occurs when $\gamma_{\text{deg}} \approx \gamma/3$.

7 Feedback in proliferation rate when decreasing the cell cycle noise

In the main article, we did not consider the effects of randomness in the duration of the cell cycle mainly for simplicity on the theoretical approach. However, it is possible to check the robustness of the theoretical conclusions effects using stochastic simulation methods.

In the proposed simulations, based on previous models [3], we consider that cells divide after crossing a certain number of stages, which we called division steps. After division, cells are reset to the first stage and increase the number of steps with a

rate proportional to the division rate defined in Equation (2) in the main text. To obtain cell cycles of approximately the same mean duration, given a number of stages N, the stage-crossing rate is given by $N\frac{\gamma}{1+kx}$. The way in which the cell cycle noise $CV^2_{\tau_d}$ changes with the total number of division steps N is presented in Fig E(A). It is possible to see that an increase in the division steps makes $CV^2_{\tau_d}$ decrease. However, the mean protein level does not show changes when $CV^2_{\tau_d}$ decreases (Fig E(B)) for both single-cell and population approaches. The protein noise shows negligible changes when decreasing $CV^2_{\tau_d}$ (Fig E(C)).

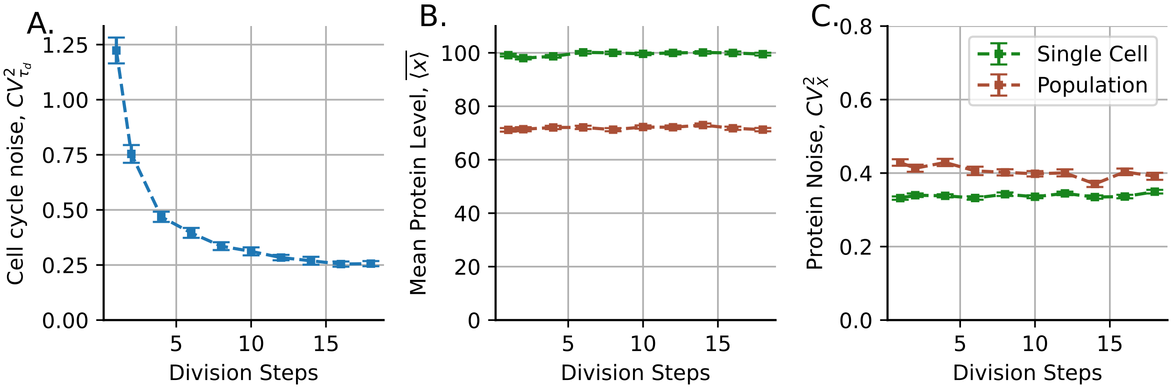

Fig E. Decreasing the noise in cell cycle duration shows negligible effects on the protein statistics: Cell division is taken to occur after passing certain amount of stages (division steps). The rate of stage crossing is proportional to the dilution rate. (**A**) Random variability of the cell cycle noise as function of the division steps. **(B)** Mean protein level for single cell (green) compared to the mean for population (brown) as the number of division steps is increased and therefore the noise in cell cycle duration decreases. **(C)** Noise in protein level as the number of division steps is increased. For all plots we set $k = 0.03$, $\beta = 10$, $\gamma = 1$. The division rate is the number of steps times the division rate (given by equation (2) in the main text). For the estimations, we use 2000 simulated replicas. Error bars represent 95% confidence intervals using bootstrapping methods.

8 Statistical properties of protein partitioning

In this appendix, we derive the statistical properties of the jumps that the protein concentration performs during partitioning.

8.1 Statistics of protein level during partitioning

First, consider a scenario in which just before cell division, the protein concentration within the cell is indicated as $x = \frac{n_d}{s_d}$, where n_d represents the number of protein molecules and s_d is the cell size. As the cell divides, its size is halved, and each daughter cell inherits a size of $\frac{s_d}{2}$. The total number of molecules, n_d, is assumed to segregate with a probability of 0.5 for each daughter. Consequently, the number of molecules in each daughter cell follows a binomial distribution with parameters n_d and 0.5.

Given these premises, the mean and variance of the protein count in each daughter cell, denoted as n_+, have the moments of the binomial distribution:

$$\langle n_+ | n_d \rangle = \frac{n_d}{2}, \quad \text{Var}(n_+ | n_d) = \frac{n_d}{4}. \tag{8.1}$$

This allows us to find the mean and variance of the protein concentration in a daughter cell:

$$\langle x_+ | n_d, s_d \rangle = \frac{\langle n_+ | n_d \rangle}{s_d/2} = \frac{n_d}{s_d} = x, \tag{8.2a}$$

$$\text{Var}(x_+ | n_d, s_d) = \frac{\text{Var}(n_+ | n_d)}{(s_d/2)^2} = \frac{n_d}{4(s_d/2)^2} = \frac{x}{s_d}. \tag{8.2b}$$

In the general case, protein partitioning may not be perfectly binomial and have complex mechanisms. To generalize expression (8.2b), let us define the statistics for the protein level after partitioning:

$$\langle x_+ | x \rangle = x, \quad \text{Var}(x_+ | x) = \varepsilon x, \tag{8.3}$$

where $\varepsilon > 0$ is a constant that reflects these effects of size scaling and complex partitioning. For the case of binomial partitioning, $\varepsilon = 1/s_d$.

8.2 Simulation of protein partitioning

During partitioning, protein levels change following the statistics shown in (8.3) with the additional constraint that $0 < x_+ < 2x$. In our simulations, we model the protein partitioning, proposing that the protein level after division follows:

$$x_+ = 2x\delta; \quad \delta \sim Beta(a, b), \tag{8.4}$$

which means that we multiply the protein before division x by two times a random variable δ which is beta-distributed with shape parameters a and b. To obtain the desired stochastic properties of x_+ as defined in (8.3), we have to satisfy $\langle\delta\rangle = 1/2$ and $\mathrm{Var}(\delta) = \varepsilon x$. Using these constraints, we obtain the values for the shape parameters of the beta distribution (8.4):

$$a = b = \frac{1}{2}\left(\frac{x}{\varepsilon} - 1\right), \quad \text{for } x > \varepsilon. \tag{8.5}$$

Due to the properties of the beta distribution, the limit $x = \varepsilon$ means that δ takes the values of 0 and 1, each with equal probability. This scenario represents the highest possible variance for any distribution confined to the interval (0,1). For a biological interpretation, if ε satisfies $\varepsilon = 1/s_d$, then $x \leq \varepsilon$ means that the progenitor cell either does not contain any protein molecules (leaving nothing to segregate) or contains exactly one. Hence, during cell division, this single molecule is inherited randomly by one of the descendants, leaving the other cell without any molecules. In our simulations, when $x \leq \varepsilon$, one descendant cell receives a protein concentration of $2x$, while its counterpart receives none.

9 Derivation of the protein steady-state mean and noise considering partitioning noise: single-cell perspective

In this appendix we present our theoretical framework for calculating the statistical moments of protein concentration, taking into account the role of molecular partitioning in gene expression. For this purpose, we use the framework of time-triggered stochastic hybrid systems (TTSHS) [4–6], which integrates the continuous dynamics of protein dilution with two families of resets: (i) cell division and (ii) protein synthesis in bursts.

To model cell division we leverage the theory of renewal processes that is a generalization of the classical Poisson process. Here the time between two successive events is an independent and identically distributed random variable following an arbitrary distribution $p_{\tau_d}(y)$, i.e., the probability

$$\mathbb{P}\{\tau_d \in (y, y + dy]\} = p_{\tau_d}(y)dy. \tag{9.1}$$

To model division events we introduce a timer τ that is set to ($\tau = 0$) when a cell is born, and increases linearly with time along the cell cycle

$$\frac{d\tau}{dt} = 1, \tag{9.2}$$

together with decay in protein concentration

$$\frac{\mathrm{d}x}{\mathrm{d}t} = -\gamma x, \tag{9.3}$$

as a per fixed dilution rate γ. Cell division events occur probabilistically with propensity (or hazard rate)

$$h(\tau) = \frac{p_{\tau_d}(\tau)}{1 - \int_0^\tau p_{\tau_d}(y)dy}. \tag{9.4}$$

More specifically, given the state of the timer τ, the probability of a division event occurring in the next infinitesimal time interval $(t, t+dt]$ is $h(\tau)dt$. Whenever this division event occurs, the timer and concentration are reset as

$$x \mapsto x_+ \quad \tau \mapsto 0, \tag{9.5}$$

with x_+ representing the protein concentration in one of the randomly-chosen daughters in the single-cell perspective. The statistical properties of x_+ were described in Section 8.1. The case of Poisson process is recovered if p_{τ_d} is precisely an exponential distribution with mean $\langle\tau_d\rangle$, then as per (9.4) the cell division propensity $h(\tau) = 1/\langle\tau_d\rangle$ would be a constant. Note that in this model formulation τ is a stochastic process and the steady-state expected value of any generalised hazard-rate satisfies:

$$\overline{\langle h(\tau)\rangle} = \frac{1}{\langle\tau_d\rangle}, \tag{9.6}$$

as shown in [5]. Having explained the timing of cell division, we next describe protein synthesis events occurring in stochastic bursts. Synthesis events occur as per a constant propensity λ, and each event increases the protein concentration as per the reset

$$x \to x + b \tag{9.7}$$

where b an independent and identically distributed random variable following an exponential distribution with mean $1/\beta$. This model of protein synthesis can be further generalized where both the time interval between two successive bursts events and the burst size b follow arbitrary distributions [7]. In summary, the deterministic dynamics (9.2)-(9.3) together with cell division and protein bursting events with corresponding resets (9.5) and (9.7), respectively, define a time-triggered stochastic hybrid systems (TTSHS) which is itself a special class of PDMPs.

We refer the reader to [4, 5] for details on deriving time evolution of the statistical moments of TTSHS state space. Using the statistical properties of x_+ in (8.3), the time evolution of the first- and second-order moments of the protein concentration follow the system of differential equations

$$\frac{d\langle x\rangle}{dt} = \lambda\beta - \gamma\langle x\rangle \tag{9.8a}$$

$$\frac{d\langle x^2\rangle}{dt} = 2\lambda\langle x\rangle\beta + \lambda\langle b^2\rangle - 2\gamma\langle x^2\rangle + \varepsilon\langle h(\tau)x\rangle. \tag{9.8b}$$

Using the fact that at steady-state (see Theorem 1 in [5])

$$\overline{\langle h(\tau)x\rangle} = \frac{\overline{\langle x\rangle}}{\langle \tau_d\rangle}, \tag{9.9}$$

and $\langle b^2\rangle = 2\beta^2$, $\gamma\langle\tau_d\rangle = \ln 2$, solving (9.8) at steady state yields

$$\overline{\langle x\rangle} = \frac{\lambda\beta}{\gamma}, \quad \overline{\langle x^2\rangle} = \overline{\langle x\rangle}^2 + \overline{\langle x\rangle}\beta + \frac{\varepsilon\overline{\langle x\rangle}}{2\gamma\langle\tau_d\rangle}, \tag{9.10}$$

which results in the following expression for concentration noise

$$\mathrm{CV}_x^2 = \frac{1}{\overline{\langle x\rangle}}\left(\frac{\varepsilon}{2\ln 2} + \frac{\langle b^2\rangle}{2\langle b\rangle^2}\right) = \frac{1}{\overline{\langle x\rangle}}\left(\frac{\varepsilon}{2\ln 2} + \beta\right). \tag{9.11}$$

10 Partitioning noise with deterministic cell cycle duration

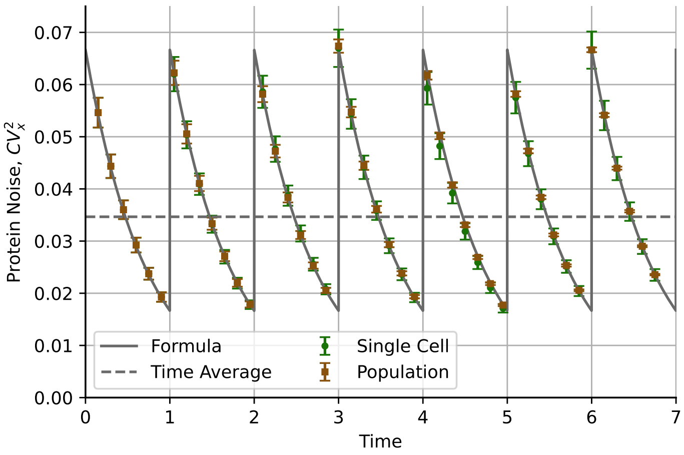

Fig F. Protein concentration noise in single-cell and population perspectives follows the same dynamics for fixed cell-cycle duration. The line represents the protein noise calculated using (10.11). The scatter-plot shows the results of simulations (green circles: single-cell, brown squares: population). Parameters: $\gamma = \ln 2$, $\lambda\beta = 20\gamma$, $\varepsilon = 1$ $\tau_d = 1$, 5000 simulation replicas.

In this appendix, we solve the moments of protein concentration from the single-cell and population perspectives when the cell cycle has a deterministic duration. As a final result, we show that the protein concentration noise is the same in both perspectives.

10.1 Noise at single cell level

First, we solve the moment dynamics for the single-cell perspective. We neglect protein bursting and therefore assume that protein concentration evolves deterministically as per (15). In this way, protein concentration trajectories consist of continuous dynamics interrupted by random-size jumps that represent partitioning during division. These divisions occur with a period of deterministic duration τ_d and therefore we can solve the ODE for protein concentration (15) applying periodic boundary conditions. This means that the system has the same properties after each multiple of τ_d.

We define the timer τ to track the time since the previous division following $\frac{d\tau}{dt} = 1$. During the cell cycle $\tau \in [0, \tau_d)$, the protein concentration evolves as per (15) which has the solution:

$$x(\tau) = x_+ e^{-\gamma\tau} + \frac{\lambda\beta}{\gamma}(1 - e^{-\gamma\tau}), \quad 0 \le \tau \le \tau_d, \tag{10.1}$$

where we have used the property (9.5) that, during division, protein levels jump from $x(\tau_d)$ to the random variable x_+ with unknown moments $\langle x_+\rangle$ and $\langle x_+^2\rangle$. Using the periodic boundary conditions, and considering that during division the mean concentration does not change, we have:

$$\langle x(0)\rangle = \langle x_+\rangle = \langle x(\tau_d)\rangle. \tag{10.2}$$

Taking the expected value of (10.1) and using the boundary conditions (10.2), we solve for $\langle x_+\rangle$:

$$\langle x_+\rangle = \frac{\lambda\beta}{\gamma}, \tag{10.3}$$

which also solves the mean concentration as function of τ:

$$\langle x(\tau)\rangle = \frac{\lambda\beta}{\gamma}, \tag{10.4}$$

which is consistent with the fact that the division does not change the mean concentration level. To obtain the variance of x over time, we find the second moment $\langle x^2(\tau)\rangle$. Since the protein trajectory for given x_+ is deterministic, the square of the protein level follows:

$$x^2(\tau) = \left(x_+ e^{-\gamma\tau} + \frac{\lambda\beta}{\gamma}(1 - e^{-\gamma\tau})\right)^2, \tag{10.5}$$

which has the expected value given τ:

$$\langle x^2(\tau)\rangle = \langle x_+^2\rangle e^{-2\gamma\tau} + \left(\frac{\lambda\beta}{\gamma}\right)^2 (1 - e^{-2\gamma\tau}). \tag{10.6}$$

Next step is to apply the boundary conditions. During cell division, this is, when time is a multiple of τ_d, the protein level jumps from $x(\tau_d)$ to x_+ and the variance increases as:

$$\begin{aligned}\mathrm{Var}(x_+) &= \mathrm{Var}(x)|_{\tau=\tau_d} + \varepsilon\langle x\rangle|_{\tau=\tau_d}\\ &= \mathrm{Var}(x(\tau_d)) + \varepsilon\frac{\lambda\beta}{\gamma}.\end{aligned} \tag{10.7}$$

Notice that $\mathrm{Var}(x_+) - \mathrm{Var}(x(\tau_d)) = \langle x_+^2\rangle - \langle x^2(\tau_d)\rangle$ because the averages are identical $\langle x\rangle|_{\tau=\tau_d} = \langle x_+\rangle$. Therefore, we conclude that:

$$\langle x_+^2\rangle = \langle x^2(\tau_d)\rangle + \varepsilon\frac{\lambda\beta}{\gamma}. \tag{10.8}$$

It is possible to replace $\langle x^2(\tau_d)\rangle$ from equation (10.6) into (10.8) and solve for $\langle x_+^2\rangle$ to obtain:

$$\langle x_+^2\rangle = \left(\frac{\lambda\beta}{\gamma}\right)\frac{\varepsilon}{(1 - e^{-2\gamma\tau_d})} + \left(\frac{\lambda\beta}{\gamma}\right)^2. \tag{10.9}$$

Substitution of this second moment of x_+ into (10.6) yields:

$$\langle x^2(\tau)\rangle = \left(\frac{\lambda\beta}{\gamma}\right)\frac{\varepsilon e^{-2\gamma\tau}}{(1 - e^{-2\gamma\tau_d})} + \left(\frac{\lambda\beta}{\gamma}\right)^2. \tag{10.10}$$

Using also $\langle x(\tau)\rangle$, it is possible to obtain the protein noise throughout the cell cycle:

$$\left(\mathrm{CV}_x^2(\tau)\right)_{SC} = \left(\frac{\gamma}{\lambda\beta}\right)\frac{\varepsilon e^{-2\gamma\tau}}{(1 - e^{-2\gamma\tau_d})}. \tag{10.11}$$

Fig F presents the trajectory of $\left(\mathrm{CV}_x^2(\tau)\right)_{SC}$ using (10.11); the comparison with simulations shows a relatively good match.

10.2 Noise at population level

In this subsection, we demonstrate that the noise at the population level coincides with the noise at the single-cell perspective. From the population perspective, after division at $\tau = 0^+$, we continue to follow both daughter cells. While one daughter cell inherits the protein level x_+, the other inherits the level $2\langle x(\tau_d)\rangle - x_+$. This is because the mean concentration of both cells just after the division is the same before the division, that is $\langle x(\tau_d)\rangle$. After a timer $\tau \in [0, \tau_d)$, the protein concentration in the first daughter is presented in (10.1). The second daughter follows the dynamics:

$$x(\tau)_{2^{nd} daughter} = (2\langle x(\tau_d)\rangle - x_+)e^{-\gamma\tau} + \frac{\lambda\beta}{\gamma}(1 - e^{-\gamma\tau}). \tag{10.12}$$

Using the property $\langle x_+ \rangle = \langle x(\tau_d) \rangle$, we obtain that the mean concentration for both daughters at equilibrium are identical:

$$\langle x(\tau)_{1^{st} daughter} \rangle = \langle x(\tau)_{2^{nd} daughter} \rangle = \lambda\beta/\gamma. \tag{10.13}$$

To obtain the dynamics of the second-order moment for the second daughter, we take the square of the equation (10.12) and take the average:

$$\begin{aligned}\langle x^2(\tau)_{2^{nd} daughter} \rangle &= \left\langle \left(2\langle x(\tau_d)\rangle - x_+)e^{-\gamma\tau}\right)^2 \right\rangle + \left\langle \left(\frac{\lambda\beta}{\gamma}(1-e^{-\gamma\tau})\right)^2 \right\rangle \\ &+ \left\langle 2\left(2\langle x(\tau_d)\rangle - x_+)e^{-\gamma\tau}\right)\left(\frac{\lambda\beta}{\gamma}(1-e^{-\gamma\tau})\right) \right\rangle \\ &= \langle x_+^2 \rangle e^{-2\gamma\tau} + \left(\frac{\lambda\beta}{\gamma}\right)^2 (1-e^{-2\gamma\tau}),\end{aligned} \tag{10.14}$$

where we have used $\langle x_+ \rangle = \langle x(\tau_d) \rangle = \frac{\lambda\beta}{\gamma}$. Notice that equation (10.14) is the same as (10.6) which has solution (10.10):

$$\langle x(\tau)^2_{1^{st} daughter} \rangle = \langle x(\tau)^2_{2^{nd} daughter} \rangle = \left(\frac{\lambda\beta}{\gamma}\right) \frac{\varepsilon e^{-2\gamma\tau}}{(1-e^{-2\gamma\tau_d})} + \left(\frac{\lambda\beta}{\gamma}\right)^2. \tag{10.15}$$

The first and second order moments of both daughters are identical, so that the $\left(\mathrm{CV}^2_x(\tau)\right)_{Pop}$ during the cell cycle $\tau \in [0, \tau_d)$ can be obtained as follows:

$$\begin{aligned}\left(\mathrm{CV}^2_x(\tau)\right)_{Pop} = \frac{\left(\mathrm{Var}^2_x(\tau)\right)_{Pop}}{\langle x(\tau)\rangle^2_{Pop}} &= \frac{\frac{1}{2}\sum_{i=1}^{i=2}\left(\langle x(\tau)^2_{i^{th} daughter}\rangle - \langle x(\tau)_{i^{th} daughter}\rangle^2\right)}{\frac{1}{2}\sum_{i=1}^{i=2}\langle x(\tau)_{i^{th} daughter}\rangle^2} \\ &= \left(\frac{\gamma}{\lambda\beta}\right)\frac{\varepsilon e^{-2\gamma\tau}}{(1-e^{-2\gamma\tau_d})},\end{aligned} \tag{10.16}$$

which is the same as the single cell noise given by (10.11). Consequently, it can be shown that after the nth division, the noise level within the population is equal to that of single cell, i.e.,

$$\left(\mathrm{CV}^2_x(\tau)\right)_{Pop} = \left(\mathrm{CV}^2_x(\tau)\right)_{SC} = \mathrm{CV}^2_x(\tau).$$

This result is verified using the simulations, which is shown in Fig F. It can be shown that with the parameters $\gamma = \ln 2/\tau_d$ and $\tau_d = 1$, the time-averaged CV^2_x is given by,

$$\frac{1}{\tau_d}\int_0^{\tau_d} \mathrm{CV}^2_x(\tau)d\tau = \frac{1}{\langle x \rangle}\left(\frac{\varepsilon}{2\ln 2}\right), \tag{10.17}$$

which corresponds to the dashed horizontal line in Fig F.

11 Simulation algorithm for estimating the protein statistics in SC and population model

In this appendix, we present the basis of the simulation algorithms used in the main article. The main idea of Algorithm 1 is to produce two time intervals for the next reactions and select the minimum. The reaction related to that minimum time will be chosen to happen. The first reaction corresponds to the protein burst, and the second is the division event. The main difficulty in the simulation relative to other standard methods such as the classical Gillespie's algorithm [8] is that the division time is not exponentially distributed, and therefore division is not a memoryless process. This means that each cell has to track the time left for division. In addition, when the population perspective is considered, the number of cells in the population increases during each division. To address this, we designed an agent-based algorithm that models protein levels within each cell over time.

The algorithm has the goal of estimating the protein level x^i of cells in the population. Here, the superscript i represents the i-th cell of the population. When the **single-cell perspective** is considered, only one descendant is randomly chosen to inherit a beta-distributed protein level during each division, so the population corresponds to one cell. Otherwise, in **population model**, a new cell is added to the population after each division, the number of cells in the population grows each division.

The process starts with setting the initial conditions. For each replica, we start the colony progenitor ($i = 1$) with a given protein level x^1. Simultaneously, we set the maximum time to end the simulation T. Each cell has two additional variables: the time to the next burst τ^i_b and the time to division τ^i_d. While τ^i_b is exponentially distributed with mean $\langle \tau^i_b \rangle = 1/\lambda$, τ^i_d is considered as gamma distributed with shape and scale parameters selected, so that this variable has mean $\langle \tau_d \rangle$ and variability $CV^2_{\tau_d}$. In some cases we consider gene expression as non-bursty, this means that the burst does not occur. This can be done by considering $\tau^i_b \to \infty$ and choosing always the division.

During each iteration, the minimum time τ_{min} is selected among all τ_d^i and τ_b^i, and the respective reaction occurs in the i-th cell. Before the reaction, all τ_d^i and τ_b^i decrease by τ_{min}. Simultaneously, the protein level of all cells evolve following the differential equation:

$$\frac{dx^i}{dt} = g(x^i, t), \quad (11.1)$$

where the function $g(x, t)$ depends on whether the gene expression is bursty or not. For a bursty protein synthesis the protein dilutes as $g(x, t) = -\gamma x$. In a non-bursty case, protein evolves as $g(x, t) = \lambda\beta - \gamma x$, and we reassign $\tau_b^i = \infty$ in Algorithm 1, so that the division is the only possible event.

We denote by n the cell that is the one with the corresponding minimum time. If τ_b^n is that minimum, the protein burst is set to happen; otherwise, the division occurs. During the burst, the protein level increases by a jump with size drawn from an exponential distribution with mean β. During division, the protein level is reset to a new variable x_+ with mean $\langle x_+ \rangle = x$ and variance $\text{Var}(x) = \varepsilon x$ as explained in Section 8.2.

When the population perspective is chosen, during division, a new cell is added to the population. While, randomly, one cell inherits a protein level x_+ with the statistics explained before, the other cells inherit a level $2x - x_+$. This ensures that the mean protein level across both daughter cells is maintained at x. For single cell model, only one of the descendants is randomly chosen to inherit a protein level x_+, maintaining the population size constant at one cell.

This simulation is performed across multiple populations, involving 5000 replicates. To estimate the moments of the distribution, for the single-cell perspective, we calculate the average across 5000 cells, one from each colony, at the end of the simulation. For the population perspective, statistical estimates are derived from all cells across 5000 colonies, without distinguishing between lineages.

12 Pop and SC difference for the adder division

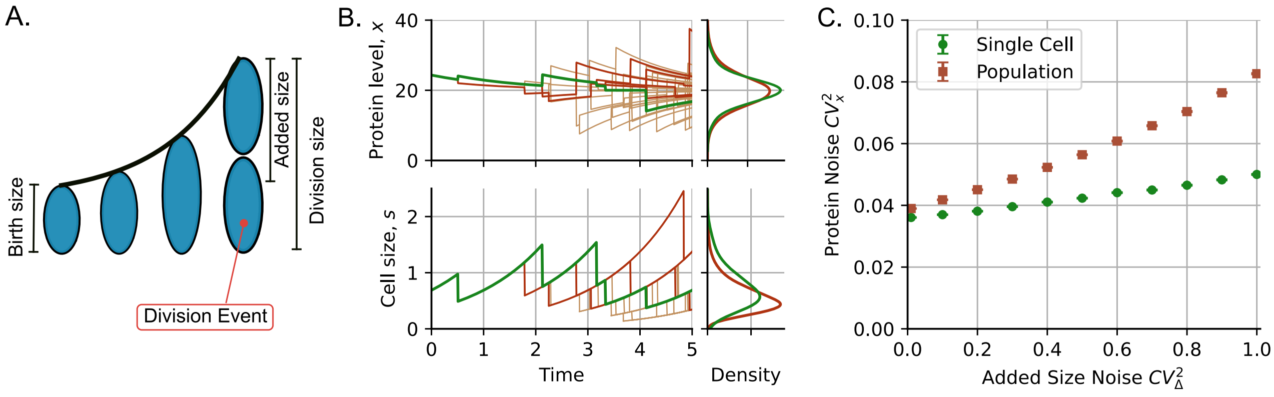

Fig G. Adder-based cell size homeostasis causes differences in concentration noise levels between the population and single-cell frameworks. (**A**) Cell size dynamics following adder principle. After division, a cell has a given birth size, grows exponentially, and divides once it has expanded by added size. The added size is an independent random variable with a given noise CV_Δ^2. (**B**) Trajectories of protein level (top) and cell size (bottom) in a sample population. Green lines represent the protein levels of a single cell, dark brown are the trajectories of the descendant cells and other trajectories correspond to remaining cells in the colony. (**C**) Comparison of noise in protein levels as a function of noise in added size CV_Δ^2. Green scatter plots show single-cell statistics and brown ones are for population. While the noise at the single-cell level changes weakly with the noise in added size, there is a greater change at the population level. Parameters of protein concentration: $\lambda\beta = 20$, $\gamma = \ln(2)$, $\varepsilon = 1$. Parameters of cell size: $\langle s_b \rangle = 0.5$, $\langle s_d \rangle = 1$, Δ is drawn from gamma distribution with $\langle \Delta \rangle = 0.5$. Single-cell statistics are based on the simulation of 50000 individual cells, population statistics are derived from 1000 colonies after approximately six divisions.

We consider that cell size increases exponentially over time with divisions following the *adder* strategy [9] – cells divide after adding, on average, a fixed cell size called *added size*. This added size is presented in Fig G(A) as the difference between the cell size at birth and the cell size at division.

During the cell cycle the cell size $s(t)$ grows exponentially:

$$\frac{ds}{dt} = \gamma s, \quad (12.1)$$

with the same growth rate γ as the protein dilution rate. The protein concentration evolves deterministically according to (15) with constant synthesis and dilution rates. During each cell division, the protein partitioning follows the method presented in Appendix 8. Then, according to (8.3), if the cell has concentration x and size s_d at the end of its life cycle, the mean protein concentration in the new cells is equal to x, with variance x/s_d. In Fig G(B), we show the cell protein concentration dynamics (top) and the cell size dynamics (bottom).

During a cell cycle, cells add a size denoted by Δ. For simulations, we assume that Δ is a random independent variable that follows a gamma distribution with mean $\langle \Delta \rangle = 0.5$ and noise CV_Δ^2. This choice of $\langle \Delta \rangle$ is set so that the mean size at division $\langle s_d \rangle = 1$, making the results of *adder* comparable to those of *timer* in the main text, following $\varepsilon = 1/\langle s_d \rangle = 1$.

To obtain the cell cycle duration τ_d for each cell, we register its birth size s_b, and then the size at the end of that cycle is $s_b + \Delta$. Given the exponential cell growth, we have $s_b + \Delta = s_b e^{\gamma \tau_d}$, which we use to obtain the cell cycle duration as follows:

$$\tau_d = \frac{1}{\gamma} \ln \left(\frac{s_b + \Delta}{s_b} \right). \tag{12.2}$$

Note that the duration of the cell cycle τ_d is now a variable that depends on s_b. This means that, unlike the *timer* approach considered in the text, τ_d is no longer an independent variable.

For simulation, each cell x^i in a colony has parameters: protein level x_i, cell size s_i, added size Δ^i, time for next burst τ_b^i drawn from the exponential distribution, and cell cycle duration τ_d^i estimated using (12.2). Then Algorithm 2 shows that based on these parameters each cell colony evolves as follows. The set of all τ_d^i and τ_b^i over the cells in the colony defines all possible reaction times. During each iteration, we select the minimum of those times, perform the associated reaction, and allow the system to evolve during that minimum time. The evolution of the colony includes the exponential growth of cells at a rate γ with the subsequent dilution of proteins at the same rate. If the selected reaction corresponds to a burst, the protein level of the cell increases by an exponentially distributed quantity with mean β. If the reaction is a division event, the protein level is perturbed, as explained in Appendix 8, and, for population level, another cell is added to the colony. The iteration continues until the maximum time T is reached.

Given that there is no simple way to measure the noise in τ_d because the duration of the cycle is defined by Δ instead, we simulate CV_x^2 as a function of the noise in the added cell size CV_Δ^2. A comparison of the level of noise protein CV_x^2 in the population and the single-cell perspective is presented in Fig G(C). We observe that with the *adder* method of cell division, the difference in protein noise level between the population and single cell is similar to the scenario where cell division follows the *timer* strategy (Fig 3C in the main text). However, protein noise for a single cell has a slight increase with CV_Δ^2. This is because the variance of the protein level after partitioning is a function of s_d and the noise in this variable increases with CV_Δ^2.

13 Feedback in frequency. Identical protein distribution in SC and population model: Proof

When we generalize the burst frequency to a function $\lambda(x)$ of the protein concentration, the time-evolution of the protein concentration pdf $p_{SC}(x, t)$ is governed by the Chapman-Kolmogorov equation:

$$\frac{\partial p_{SC}(x,t)}{\partial t} = \frac{\partial}{\partial x}(\gamma x p_{SC}(x,t)) + \int_0^x b(x-y)\,\lambda(y) p_{SC}(y)\,\mathrm{d}x \\ - \lambda(x) p_{SC}(x), \tag{13.1}$$

where $b(x) = \mathrm{e}^{-x/\beta}/\beta$ is the probability density function of the exponential distribution. Note that there is no feedback in dilution in (13.1).

To find the stationary distribution, we write it as a probability conservation equation. It is done by gathering the last two terms of (13.1) as per Leibniz integral rule into the following derivative:

$$\int_0^x b(x-y)\,\lambda(y) p_{SC}(y)\,\mathrm{d}y - \lambda(x) p_{SC}(x,t) = -\frac{\mathrm{d}}{\mathrm{d}x} \int_0^x \bar{\mathrm{B}}(x-y)\,\lambda(y) p_{SC}(y)\,\mathrm{d}y\,, \tag{13.2}$$

where $\bar{\mathrm{B}}(x)$ is the complementary cumulative distribution function (ccdf) corresponding to $b(x)$, i.e., $\bar{\mathrm{B}}(x) = \mathrm{e}^{-x/\beta}$.

The steady state of the system implies that distribution does not change over time, i.e., $\partial p_{SC}(x,t)/\partial t = 0$. Combining (13.1) and (13.2), and integrating the result, we obtain:

$$\gamma x p_{SC}(x) = \left(\bar{B} * \tilde{p}\right)(x), \tag{13.3}$$

where an auxiliary function $\tilde{p}(y) = \lambda(y) p_{SC}(y)$ is convolved with the burst-size ccdf. The volterra integral equation (13.3) can be solved by standard methods.

We proceed with the model of the population, where the lifecycle of each cell is identical to one described at the beginning of the chapter. Its composition is given by the function $h(x, t)$ – number of cells with given concentration x at the time t. The time evolution of $h(x, t)$ is described by the population balance equation:

$$\frac{\partial h(x,t)}{\partial t} = \frac{\partial}{\partial x}(\gamma x h(x,t)) + \gamma h(x,t) - \frac{\partial}{\partial x}\left(\int_0^x \bar{\mathrm{B}}(x-y)\,\lambda(y) p(y)\,\mathrm{d}y\right), \tag{13.4}$$

which differs from the Chapman-Kolmogorov equation (13.1) by the inclusion of the population growth term $\gamma h(x, t)$.

However, in the absence of the feedback in dilution, we have exponentially distributed lifespans. In this case, the population growth rate depends only on the division frequency, i.e., the principal eigenvalue satisfies $\mu = \gamma$. By substitution, we can prove that solutions of the PBE (13.4) satisfy

$$h(x,t) = p_{SC}(x,t) e^{\gamma t},$$

where $p_{SC}(x, t)$ solves the master equation (13.1). From it follows, in particular, that

$$h(x,t) \sim p_{SC}(x) e^{\gamma t}, \quad t \to \infty,$$

where $p_{SC}(x)$ solves the stationary problem for the single-cell perspective (13.3). Hence, the stationary distributions of the protein concentration for single-cell perspective $p_{SC}(x)$ and population perspective $p_{Pop}(x)$ are identical in the absence of the dilution feedback.

Data: $\mathbb{X} = \{x^1\}, T$
Result: $\mathbb{X} = \{x^1, \cdots, x^N\}$
$\tau_d^1 \sim gamma(\langle \tau_d \rangle, CV^2_{\tau_d})$;
$\tau_b^1 \sim Exponential(\langle \tau_b \rangle)$;
$N = |\mathbb{X}| = 1$;
$\tau_{min} = \min_{\forall i \in \{1\}}(\tau_d^i, \tau_b^i)$;
$n = \mathrm{argmin}_{\forall i \in \{1\}}(\tau_d^i, \tau_b^i) = 1$;
$t = \tau_{min}$;
$\tau' = 0$;
if $\tau_{min} > T$ **then**
  $x^1 = \int_0^T g^1(u)du$
else
  while $t < T$ **do**
    for $i = 1;\ i \leq N;\ i = i + 1$ **do**
      $x^i = x^i + \int_0^{t-\tau'} g^i(u)du$;
      $\tau_b^i = \tau_b^i - (t - \tau')$;
      $\tau_d^i = \tau_d^i - (t - \tau')$;
    end
    if $\tau_{min} == \tau_b^n$ **then**
      $b \sim Exponential(\beta)$;
      $x^n = x^n + b$;
      $\tau_b^n \sim Exponential(\langle \tau_b \rangle)$;
    else
      $\delta \sim Beta\left(\frac{x^n/\varepsilon - 1}{2}, \frac{x^n/\varepsilon - 1}{2}\right)$;
      $x_+ = 2x^n\delta$;
      if *Population* **then**
        $x^{N+1} = 2x^n - x_+$;
        $\tau_d^{N+1} \sim gamma(\langle \tau_d \rangle, CV^2_{\tau_d})$;
        $\tau_b^{N+1} \sim Exponential(\langle \tau_b \rangle)$;
        $N = |\mathbb{X}| = N + 1$;
      else
        $N = 1$;
      end
      $x^n = x_+$;
      $\tau_d^n \sim gamma(\langle \tau_d \rangle, CV^2_{\tau_d})$;
    end
    $\tau_{min} = \min_{\forall i \in \{1, \cdots, N\}}(\tau_d^i, \tau_b^i)$;
    $n = \mathrm{argmin}_{\forall i \in \{1, \cdots, N\}}(\tau_d^i, \tau_b^i)$;
    $\tau' = t$;
    $t = t + \tau_{min}$;
  end
  for $i = 1;\ i \leq N;\ i = i + 1$ **do**
    $x^i = x^i + \int_{\tau'}^T g^i(u)du$;
  end
end

Algorithm 1: Given the initial protein concentration x_1, and the time to complete the simulation T, we obtain the end of the simulation the array of protein concentrations at $\mathbb{X} = \{x^1, \cdots, x^N\}$ for the N cells. If the simulation is done for the population perspective, the algorithm saves a new descendant cell every time a cell in the population divides; for the single-cell perspective all descendants are ignored.

Data: $\mathbb{X} = \{x^1\}, \mathbb{S} = \{s^1\}, T$
Result: $\mathbb{X} = \{x^1, \cdots, x^N\}, \mathbb{S} = \{s^1, \cdots, s^N\}$
$\Delta^1 \sim gamma(\langle \Delta \rangle, CV^2_\Delta)$;
$\tau_d^1 = \frac{1}{\gamma}\ln\left(\frac{s^1 + \Delta}{s^1}\right)$;
$\tau_b^1 \sim Exponential(\langle \tau_b \rangle)$;
$N = |\mathbb{X}| = 1$;
$\tau_{min} = \min_{\forall i \in \{1\}}(\tau_d^i, \tau_b^i)$;
$n = \mathrm{argmin}_{\forall i \in \{1\}}(\tau_d^i, \tau_b^i) = 1$;
$t = \tau_{min}$;
$\tau' = 0$;
if $\tau_{min} > T$ **then**
  $x^1 = \int_0^T g^1(u)du$
else
  while $t < T$ **do**
    for $i = 1;\ i \leq N;\ i = i + 1$ **do**
      $x^i = x^i + \int_0^{t-\tau'} g^i(u)du$;
      $s^i = s^i e^{\gamma(t-\tau')}$;
      $\tau_b^i = \tau_b^i - (t - \tau')$;
      $\tau_d^i = \tau_d^i - (t - \tau')$;
    end
    if $\tau_{min} == \tau_b^n$ **then**
      $b \sim Exponential(\beta)$;
      $x^n = x^n + b$;
      $\tau_b^n \sim Exponential(\langle \tau_b \rangle)$;
    else
      $\varepsilon = 1/s^n$;
      $\delta \sim Beta\left(\frac{x^n/\varepsilon - 1}{2}, \frac{x^n/\varepsilon - 1}{2}\right)$;
      $x_+ = 2x^n\delta$;
      $s_+ = s^n/2$;
      if *Population* **then**
        $x^{N+1} = 2x^n - x_+$;
        $s^{N+1} = s_+$;
        $\Delta^{N+1} \sim gamma(\langle \Delta \rangle, CV^2_\Delta)$;
        $\tau_d^{N+1} = \frac{1}{\gamma}\ln\left(\frac{s_+ + \Delta}{s_+}\right)$;
        $\tau_b^{N+1} \sim Exponential(\langle \tau_b \rangle)$;
        $N = |\mathbb{X}| = N + 1$;
      else
        $N = 1$;
      end
      $x^n = x_+$;
      $s^n = s_+$;
      $\Delta^n \sim gamma(\langle \Delta \rangle, CV^2_\Delta)$;
      $\tau_d^n = \frac{1}{\gamma}\ln\left(\frac{s_+ + \Delta}{s_+}\right)$;
    end
    $\tau_{min} = \min_{\forall i \in \{1, \cdots, N\}}(\tau_d^i, \tau_b^i)$;
    $n = \mathrm{argmin}_{\forall i \in \{1, \cdots, N\}}(\tau_d^i, \tau_b^i)$;
    $\tau' = t$;
    $t = t + \tau_{min}$;
  end
  for $i = 1;\ i \leq N;\ i = i + 1$ **do**
    $x^i = x^i + \int_{\tau'}^T g^i(u)du$;
    $s^i = s^i e^{\gamma(T-\tau')}$;
  end
end

Algorithm 2: Simulation algorithm for cell proliferation following the *adder* division strategy.

References

[1] M. Abramowitz, I. A. Stegun, and R. H. Romer, “Handbook of mathematical functions with formulas, graphs, and mathematical tables,” 1988.

[2] N. Friedman, L. Cai, and X. S. Xie, “Linking stochastic dynamics to population distribution: an analytical framework of gene expression,” *Physical review letters*, vol. 97, no. 16, p. 168302, 2006.

[3] C. Nieto, C. A. Vargas-García , J. M. Pedraza, A. Singh, “Mechanisms of cell size regulation in slow-growing *Escherichia coli* cells: Discriminating models beyond the adder,” npj Systems Biology and Applications. 2024;10(1):61.

[4] Z. Vahdat, Z. Xu, and A. Singh, “Modeling protein concentrations in cycling cells using stochastic hybrid systems,” *IFAC-PapersOnLine*, vol. 54, no. 9, pp. 521–526, 2021.

[5] M. Soltani and A. Singh, “Moment-based analysis of stochastic hybrid systems with renewal transitions,” *Automatica*, vol. 84, pp. 62–69, 2017.

[6] M. Soltani and A. Singh, “Moment analysis of linear time-varying dynamical systems with renewal transitions,” *SIAM Journal on Control and Optimization*, vol. 57, pp. 2660–2685, 2019.

[7] T. Jia and R. V. Kulkarni, “Intrinsic noise in stochastic models of gene expression with molecular memory and bursting,” *Physical review letters*, vol. 106, no. 5, p. 058102, 2011.

[8] D. T. Gillespie, “A general method for numerically simulating the stochastic time evolution of coupled chemical reactions,” *Journal of computational physics*, vol. 22, no. 4, pp. 403–434, 1976.

[9] S. Taheri-Araghi, S. Bradde, J. T. Sauls, N. S. Hill, P. A. Levin, J. Paulsson, M. Vergassola, and S. Jun, “Cell-size control and homeostasis in bacteria,” *Current Biology*, vol. 25, pp. 385–391, 2015.
