# Supplementary material for: Stochastic gene expression in proliferating cells: Differing noise intensity in single-cell and population perspectives
Source: PLoS Comput Biol. 2025 Jun 10;21(6):e1013014. doi: 10.1371/journal.pcbi.1013014 (PMC12151482; doi:10.1371/journal.pcbi.1013014)
Supplement: Attachment — Submitted filename: PCOMPBIOL-D-24-01119-review.pdf [file pcbi.1013014.s003.pdf]

The study of Zhang et al. focuses on an understudied aspect of the stochasticity of gene expression, namely, the contribution of population-wide factors. There have been a few studies so far which investigate gene expression noise in cell populations instead of single cells, but they have used a computational approach and this is the first study to provide a theoretical background and analytical solutions for distributions of expression mean and variance in a noisy cell population. The manuscript is very well-written, the figures are satisfactory, and the important articles are referenced. I think this manuscript would be a good fit for the scope of the PLoS Computational Biology journal, when some edits are made. The code to simulate the data is publicly available, but the simulated data from which all figures and conclusions of the paper follow is not. Per the PLoS Data Policy, the data necessary to reproduce all figures must be made fully available without restriction, just like the simulator code. I would recommend this article for publication when the simulated data is made publicly available and some revisions are made. I list my recommended revisions below, ordered by importance.

1a. One of the main conclusions of this study is that population-wide expression noise is non-negligible when the focal protein affects the cell proliferative capacity, with the assumption that the focal protein is long-lived. The molecular mechanisms of cell division in both prokaryotes and eukaryotes are well understood and the molecular players (proteins - cyclins and others) are well described. How many of these proteins are long-lived vs. short-lived and actively degraded? The cell cycle necessitates the degradation of at least some of them, for example cyclin D1 has a very short half-life. Can you list examples of long-lived cell cycle proteins (if there are any), as the conclusions of this study are most relevant for these real examples? There are other modulators of cell proliferation which do not control the cell cycle directly, proteins that affect the cell size or growth factors, and would fit the protein of interest criteria. Listing some examples of genes for which the conclusions of the study stand in the discussion would elevate the relevance of the conclusions and provide real-life examples of where the population perspective is not to be ignored when studying expression noise.

1b. Also, the definition of a long-lived protein is relative to the cell-doubling time, i.e. a protein is long-lived if its half-life is significantly longer than the cell-doubling time. Since prokaryotes usually

have short cell-doubling times, would more proteins be classified as long-lived in prokaryotes? That would mean that the conclusions apply more frequently to prokaryotic than eukaryotic cells, something the readers would be interested in.

1c. Could you add a short comment on what would happen if the assumption of no degradation was relaxed? Do you expect it to change the results?

2. One of the main methods of studying stochasticity of gene expression is through single-cell measurements of protein level or sequencing of isogenic cell populations, e.g. single-cell RNA-seq, both of which would correspond to a population perspective in the text. However, results in terms of expression level distribution and variance from scRNA-seq data are often used to make claims about the expression dynamics within single cells, with the assumption that they can be equated. This study is in a unique position to study both and compare them, and I think it would be impactful to point out in the discussion when the two perspectives can be equated. This is even mentioned in the early papers studying noise (e.g. Friedman et al. 2006, cited in the manuscript) – that we are not sure if the ergodic principle from statistical physics holds for this system, in other words, whether the statistical distribution of the ensemble, the population of cells, is identical to the distribution of states over time in one process, within one cell. The scenarios in which the two perspectives yield different distribution is important, and correctly highlighted in the manuscript, but the opposite, that in many cases the distributions do not differ at all, is also insightful. Particularly since the single-cell perspective approach, to observe gene expression levels in real time, is a much more demanding experimental setup than the population approach. If one can claim that the expression distribution of one gene out of thousands of genes analyzed in an RNA-seq experiment are identical to the expression distribution of this gene over time within one cell, there is no need to do tedious massive scale GFP tagging to observe the dynamics of each individual gene in the genome.

3. I think reordering two paragraphs in the introduction would yield a better reading flow. Paragraph starting at line 33 describes the first scenario in which the population and single-cell distribution differ, then paragraph on line 44 describes the mathematical methods used in both perspectives, then paragraph on line 51 goes back to the second scenario. It would be better to describe the first scenario,

then second, then finish with the mathematical description of both (i.e. swap paragraph on line 51 and 44).

4. Line 82: why is “one-dimensional” used to describe the model of gene expression? It would be simpler to just write the telegraph model of gene expression, which is how it is commonly referred to.

5. Line 117: Text says that the case of no feedback (k = 0) is explored, while in the figure the data for weak feedback is shown (k=0.005).
