# Supplementary material for: Stochastic gene expression in proliferating cells: Differing noise intensity in single-cell and population perspectives
Source: PLoS Comput Biol. 2025 Jun 10;21(6):e1013014. doi: 10.1371/journal.pcbi.1013014 (PMC12151482; doi:10.1371/journal.pcbi.1013014)
Supplement: Attachment — Submitted filename: PLoS_Author_response_to_reviewers.pdf [file pcbi.1013014.s004.pdf]

Authors' Response to Reviews on Manuscript (PCOMPBIOL-D-24-01119) - [EMID:a091fed37246d806]

Stochastic Gene Expression in Proliferating Cells: Differing Noise Intensity in Single-Cell and Population Perspectives.

by the Manuscript's Authors
PLoS Computational Biology

***RC:** Reviewers' Comment*, **AR**: Authors' Response, ☐ Manuscript Text

The authors thank the reviewers for their feedback. Their suggestions greatly improved the quality of the article.

1. Reviewer #1

1.1. Major Comments

RC: *The current model does not account for the fact that gene expression rates are cell growth rate dependent. Are the results shown in Figure 2 affected if this dependence is included in the model?*

AR: To address the suggestion of the reviewer, we modified the model presented in the main article to perform the burst events with a frequency proportional to the protein-dependent growth rate. This can be represented by jumps in the gene product x as:

$$x \xrightarrow{\lambda(x)} x + b, \tag{R1}$$

in which $\lambda(x)$, the burst frequency, is proportional to the dilution/growth rate, i.e.

$$\lambda(x) = \frac{\lambda_{\max}}{1 + kx} = \tilde{\lambda}\gamma(x), \tag{R2}$$

where $\tilde{\lambda}$ is the maximum burst frequency $\lambda_{\max}$ normalized to the maximum dilution rate γ. In this case, the steady-state protein distribution in the single-cell model is following:

$$p_{SC}^{\lambda}(x) = \frac{1 + kx}{\beta\Gamma(\tilde{\lambda})(1 + \tilde{\lambda}k\beta)} e^{-\frac{x}{\beta}} \left(\frac{x}{\beta}\right)^{\tilde{\lambda}-1}.$$

Here and below we use superscript λ to mark distributions and moments for the model with the protein-dependent burst frequency.

The n-th steady-state raw moment of the protein concentration in the single cell follows:

$$\overline{\langle x^n \rangle}_{SC}^{\lambda} = \beta^n (\tilde{\lambda})_n \frac{1 + k\beta(\tilde{\lambda} + n)}{1 + \tilde{\lambda}k\beta},$$

where $(\tilde{\lambda})_n$ is the Pochhammer symbol, defined as $(\tilde{\lambda})_n = \Gamma(\tilde{\lambda}+n)/\Gamma(\tilde{\lambda})$, with $\Gamma(\cdot)$ being the gamma function. Calculating the mean protein level, the moment with $n = 1$, we obtain:

$$\overline{\langle x \rangle}^{\lambda}_{SC} = \tilde{\lambda}\beta \frac{1 + k\beta(\tilde{\lambda}+1)}{1+\tilde{\lambda}k\beta}. \tag{R3}$$

It is also possible to solve the steady-state protein distribution for the population perspective which results in:

$$p^{\lambda}_{POP}(x) = \frac{1+kx}{\beta\Gamma(u)(1+uk\beta)} e^{-\frac{x}{\beta}} \left(\frac{x}{\beta}\right)^{u-1}, \quad u = \frac{\tilde{\lambda}}{1+\tilde{\mu}k\beta},$$

where $\tilde{\mu}$ is the population growth rate normalized to γ and is given by:

$$\tilde{\mu} = \frac{-(1+k\beta(\tilde{\lambda}-1)) + \sqrt{(1+k\beta(\tilde{\lambda}-1))^2 + 4k\beta}}{2k\beta}.$$

The n-th steady-state raw moment of the protein concentration in the cell population is following:

$$\overline{\langle x^n \rangle}^{\lambda}_{POP} = \beta^n (u)_n \frac{1+k\beta(u+n)}{1+uk\beta}.$$

In Figure R1, we illustrate the effect of increasing the feedback intensity when the burst frequency is coupled with the dilution/proliferation rate following (R2). Figure R1A provides a reference by showing the difference between both distributions when the burst frequency is independent of the dilution rate, for a feedback strength of $k = 0.02$. In Figure R1B, using the same feedback strength ($k = 0.02$), we fix $\lambda_{\max}$ so that the mean protein level at the single-cell level is set to $\overline{\langle x \rangle}^{\lambda}_{SC} = 100$. The resultant distribution from a population perspective is also shown with these parameters.

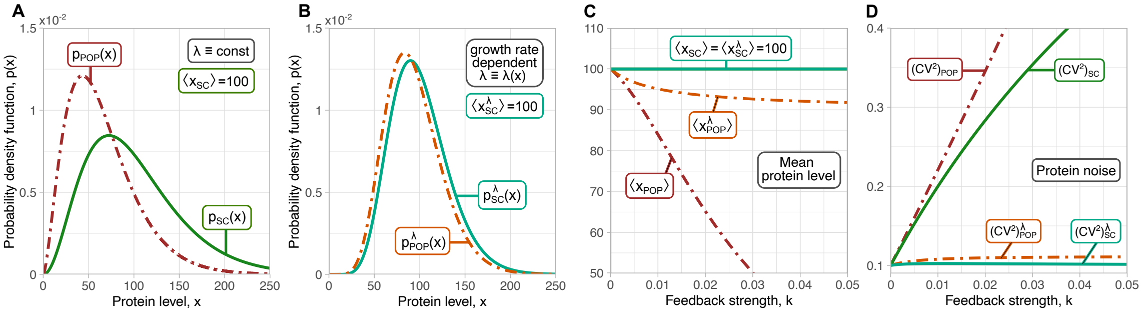

Figure R1: **Difference between single cell and population perspectives is less prominent when the transcription rate is proportional to the cell growth rate**. Comparison of protein distributions in single-cell and population perspectives as: (**A**) the burst frequency is independent from protein concentration, $\lambda_{\max}$ is chosen so that $\overline{\langle x \rangle}_{SC} = 100$ (case studied in our article); (**B**) the burst frequency is protein-dependent, $\lambda_{\max}$ is again chosen so that $\overline{\langle x \rangle}^{\lambda}_{SC} = 100$ as per (R3). (**C**) Mean protein concentration and (**D**) protein noise in both models. For all plots $k = 0.02$, $\beta = 10$, $\gamma = 1$.

Figure R1C shows how the mean protein levels diverge increasing the feedback strength. To obtain this graph, we set $\lambda_{\max}$ for each model (coupling and not coupling) so that the mean protein level in the single-cell perspective is set to $\langle x \rangle_{SC} = 100$ using (R3). The mean for the population perspective is estimated using the

respective $\lambda_{\max}$. Similarly, Figure R1D plots the noise measured using the squared coefficient of variability. This figure demonstrates that coupling the burst frequency and growth rate results in fewer differences between single-cell and population approaches. In the limit of strong feedback, the gap between the metrics in both population perspectives approximately reaches a fixed value.

These details are now in the supplementary information, whereas we added in the main article the following text:

> ...We also studied how single cell and population statistics differ considering that transcription rate is not constant but rather depends on cell growth rate. In the context in which the transcription rate is proportional to the growth rate, we observe that the feedback strength induces smaller differences in the protein moments between the mean and the protein perspectives. The mean at population level is round 10% less than the mean for single cell while the noise is essentially the same between both perspectives.

RC: *Figure 2B and C – I believe the results for the statistical properties presented are based on the analytical predictions from equations (4) (for the population) and from equations (6a-6c) (for the single cell). Can the authors show how well the model simulations results fit these predictions?*

AR: We performed simulations of both the single-cell and population models as outlined at the beginning of in paragraphs “Protein distribution for single-cell perspective" and “Protein distribution for population perspective".

For the single-cell model, the n-th moment was calculated from measurements of 3000 cells. For the population model, the statistical properties were derived from a snapshot of the population at a specific point in time, with each snapshot containing at least 5000 cells to ensure robust statistics. The results, as shown in Figure R2, showed good agreement with the analytical predictions from equations (4) and (6a-6c), indicating that the model simulations fit the theoretical values well.

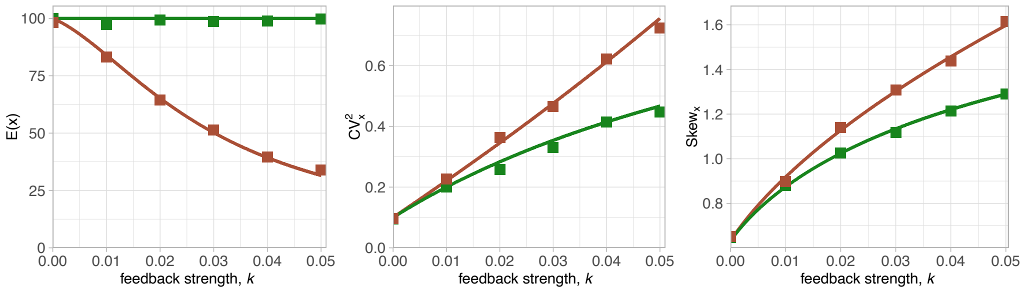

Figure R2: **Simulations and theoretical model show an excellent agreement** (**A**) Mean protein concentration, (**B**) Noise in protein concentration, (**C**) protein asymmetry as function of the feedback strength. We compare the theoretical exact formulas (solid line) with the result of stochastic simulation (scatter plot) of the two population perspectives considered: single cell (green) and population (brown). The value of λ is given by (7), so that $\overline{\langle x \rangle}_{SC} = 100$ as k increases; $\beta = 10$, $\gamma = 1$.

RC: *Line 78: ‘Protein statistics are determined based on a single lineage path over time’... in the population approach, both daughter cells are tracked, and statistics... are estimated at a fixed time point’. Given this description, I am not sure what kind of distributions are illustrated in Figure 1B. Are they single cell or population distributions?*

AR: The trajectories shown in Figure 1B (left) are sample trajectories of protein concentration in a single cell. Therefore, the distributions in Figure 1B (right) represent single-cell protein concentrations. The blue lines correspond to unregulated gene expression, while the green lines represent trajectories for the regulated dilution rates. In addition to labeling the x-axis of Figure 1B (right) as $p_{SC}(x)$ coming from the distribution at the single-cell level, we also have adjusted the caption of the figure:

> Sample trajectories of protein concentration in a single cell, along with the corresponding protein concentration distributions for both models.

RC: *Section: 'Coupling deterministic expression with partitioning error'. Can the authors clarify why only at this stage the authors decided to change the cell cycle assumption. Do the results shown in Figure 2 are robust to the relaxation of the cell cycle assumption?*

AR: The results shown in Fig. 2 are based on exact analytical derivations of the steady-state protein concentration distribution which assume exponentially-distributed cell-cycle times. We have now added material checking the robustness of these results using stochastic simulation for Erlang-distributed cell-cycle times.

In the proposed simulations, based on previous models, we consider that cells divide after crossing a certain number of stages, which we called division steps. After division, cells are reset to the first stage. Cells increase the number of steps with a rate proportional to the division rate defined in Equation (2) in the main text. To obtain cell cycles of approximately the same mean duration, given a number of stages N, the stage-crossing rate is given by $N\frac{\gamma}{1+kx}$. The way in which the cell cycle noise $CV^2_{\tau_d}$ changes with the total number of division steps N is presented in Figure R3A. It is possible to see that an increase in the division steps makes $CV^2_{\tau_d}$ decrease. However, the mean protein level does not show changes when $CV^2_{\tau_d}$ decreases (Figure R3B) for both single-cell and population approaches. The protein noise shows negligible changes when decreasing $CV^2_{\tau_d}$ (Figure R3C).

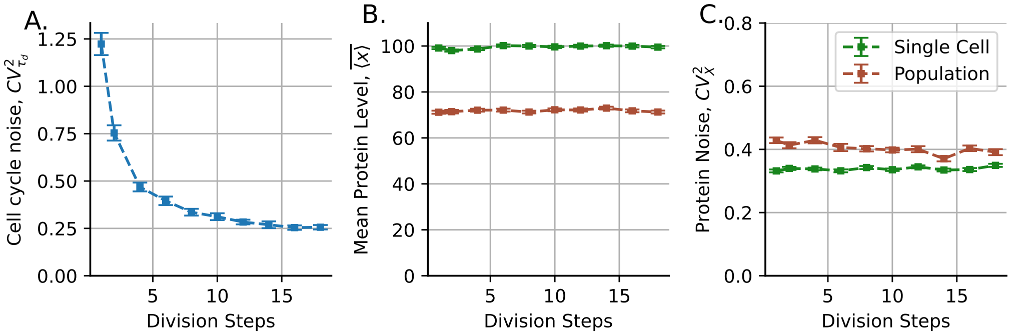

Figure R3: **Decreasing the noise in cell cycle duration shows negligible effects on the protein statistics**: Cell division is taken to occur after passing certain amount of stages (division steps). The rate of stage crossing is proportional to the dilution rate. (**A**) Random variability of the cell cycle noise as function of the division steps. **(B)** Mean protein level for single cell (green) compared to the mean for population (brown) as the number of division steps is increased and therefore the noise in cell cycle duration decreases. **(C)** Noise in protein level as the number of division steps is increased. For all plots we set $k = 0.03$, $\beta = 10$, $\gamma = 1$. The division rate is the number of steps times the division rate given by equation (2) in the main text. For the estimations, we use 2000 simulated replicas. Error bars represent 95% confidence intervals using bootstrapping methods.

In the main article we added the following text:

> We perform agent-based simulations in which the cell cycle is divided in multiple stages, reducing the noise level of the duration of the cell cycle. We found that the observed moments depend weakly on the randomness of the cell cycle and our conclusions about the difference between single-cell and population approaches are still valid.

RC: *Throughout the manuscript the authors claim to have studied/identified two scenarios for which the protein noise of the two perspectives differs. However, an extensive part of the manuscript is dedicated to the effects of randomness in cell-cycle times while keeping epson (i.e. randomness level of partitioning) constant. Could the randomness in cell-cycle times be identified as a third scenario for which protein noise differs in both perspectives (as seen in Figure 3B) ?*

AR: A key results of the paper is that in the absence of expression-growth coupling

- Deterministic cell-cycle times lead to similar noise levels in both perspectives even with $\epsilon > 0$.
- Noisy cell-cycle times lead to similar noise levels in both perspectives without partitioning noise ($\epsilon = 0$.)

Based on the second item, noisy cell-cycle times by itself are not sufficient to create differences, and have to couple it with $\epsilon > 0$ (i.e., scenario 2)

The authors thank the reviewer for pointing out this inquiry. In the main article, we add the following text:

> Observe that if there is no partitioning noise, the cell cycle noise will not affect the protein noise. This occurs since the main drivers of heterogeneity in protein levels (cycle-dependent injections of noise) are not present in that case.

RC: *Can the authors clarify if the distribution for the population perspective in Figure 3B, 4A and 4C is taken across all descendant cells (red+brown) or solely sister cells (red)? Due to the color scheme, I assume it is the latter, however, Figure 1B bottom considers both 'sister' and 'other cells' to explain the population perspective.*

AR: Thanks for noticing this lack of clarity. Now we add in the caption the following text:

> Single cell statistics are estimated over a 5000 independent individuals; population statistics are estimated using all cells of 2000 colonies (including sisters, progenitor and other cells). Statistics were calculated after 6 generations.

RC: *Line 333 reads 'at a fixed time point' (as well as in many other parts of the manuscript, e.g. line 80). However, according to the red trajectories and red distributions in Figure 3B, 4A and 4C, I assumed that the descendant cells were sampled at several fixed time points to draw the population level distribution and not solely at last time point, as depicted in Figure 1B bottom. Could the authors clarify which approach was used and perhaps update Figure 1B accordingly?*

AR: To specify the methods used for the empirical estimation of the protein statistics, we added a new subsection explaining the methods used for estimating the protein moments from simulated data. Now, this new subsection is as follows:

In our approach, we consider two main methods to estimate expected values. The first method involves taking the time-averaged value of the stochastic process over a long trajectory (as shown in Figure 1A). The second method involves taking the average of the process at a given time across different replicas (as illustrated in Figure 1B). From a mathematical perspective, when these two averaged values are equivalent, we say that the stochastic process is ergodic. Ergodicity will be used for our analytical derivations. Since it is computationally expensive to follow a proliferating population for a very long time, the results obtained from our simulations, unless otherwise specified, will be obtained using the second method. For single cell, we take the statistics over thousands of independent cells in a given time, while for population perspective, we perform the statistics over all cells in thousands of colonies in a given time.

RC: *Differences between population and single-cell distributions are found only when protein levels have feedback in the growth dynamics. Can the authors discuss how relevant is it to study this kind of proteins? Do they exist in abundance? Or is this feedback mostly due a certain protein reaching toxicity levels for the cell, thus halting cell division?*

AR: We added the following phrase with some specific examples of molecules that can be candidates for our approach:

This feedback has been observed in specific cases, including stress-response factors in bacteria and fission yeast. These stress factors regulate the transcription of other genes, thus establishing an indirect relationship between gene products and the growth rate.

Our approach can also be applied to more direct examples, such as proteins with significant toxicity levels that inhibit cell growth. This scenario is particularly relevant in the case of synthetic constructs where the gene product is non-functional to the cell. Other proteins that affect cell proliferation, such as division timing regulators, may require a more complex cell cycle-dependent modeling.

In the main text, we have added the references supporting this information.

RC: *Works have shown that a good strategy is to binomially partition the cell contents (e.g. doi:10.1371/journal.pone.0079345). Can the authors explain why binomial partition was not considered?*

AR: The binomial partitioning is not considered explicitly, because it applies specifically to discrete variables (cell number) and we study a continuous variable (concentration). When studying discrete variables, it is difficult to separate the effects of partitioning and protein bursting. The use of continuous variables, instead, helps us to study cases where there is no production-decay noise as explored in the first part of the approach. When modeling the partitioning, we considered following the statistical properties of the binomial partitioning since the first two moments of the protein concentration after the division follow the moments of the binomial distribution (the details are provided in Appendix S8). Intrinsic noise in the form of bursts is considered in the second part of the approach. To better explain this idea, we added the following paragraph to the text.

To isolate the effect of partitioning noise, we first explore an unregulated model in which gene expression evolves deterministically. Modeling protein level using a continuous variable instead of discrete values allows us to separately add specific sources of noise. Intrinsic fluctuations such as those found in discrete models are ignored and considered to be effectively captured by the bursts. First, we present the solution focusing on the partitioning noise, which will follow the binomial statistics (mean and variance) which is equivalent to the discrete counterpart. Next, we add intrinsic noise in the form

> of stochastic bursting events.

Additionally, we added the reference suggested by the reviewer.

RC: *Line 78: 'Protein statistics are determined based on a single lineage path over time'. Should it read instead: ' Protein statistics are determined based on multiple single lineage paths over time'. Since as described in Figure 4 caption, 5000 cells were studied.*

AR: The authors appreciate the reviewer's suggestion. We modified the text as proposed.

RC: *Line 314 and 315: Suggests that partitioning noise is not a form of intrinsic noise. However, line 382 calls it 'another source of intrinsic noise'. Can the authors considering clarifying what they consider to be intrinsic noise?*

AR: We thank for pointing out this inconsistency. We fixed the text and consider the burst as the effective measurement of any intrinsic noise which comes from the transcription/translation processes.

RC: *As the authors discuss, it would be of great interest to validate these predictions with empirical data. For example, one could probably make use of the data (time-lapse protein expression levels for single lineage and descendant cells) shown in Figure 2C of (DOI: 10.1126/science.1188308). Further, the same type of data at the mRNA level can also be found in (DOI:10.1371/journal.pcbi.1005174).*

AR: We thank the reader for the comment. We decided to compare the model and data in a future publication oriented to predict gene expression in more realistic scenarios using a more mechanistic approach. This article, given the simplicity of the modeling, can be used as a proof of concept.

1.2. Minor Comments

RC: *Line 64: Should it read 'Single cell and population analysis . . . ' ?*

AR: We did not notice that typo, thanks for pointing it out.

RC: *Fig 1A: What is the meaning of the four upward grey arrows? Also, are the vertical lines in the single-cell protein level illustration denoting cell division events? Are they supposed to match the division events of the root of the three?*

AR: The purpose of the arrows was to connect the top of the figure 1A with the bottom. We use the arrows as connectors to show that both, the two-dimensional plot and the colors of the lineage branch represent similar protein levels. However, it seems that the arrows were misleading. We decided to delete the arrows and just explain explicitly the connection between both figures. Therefore, we add this phrase:

> The color intensity can be related to a two-dimensional graph of protein level over time (Figure 1A top).

With respect to the vertical lines, they correspond to the grid of time. Since we did not properly label the axis once the idea was to show schematically the phenomena, we delete those lines.

RC: *Table 1: Table captions should be on top of the table*

AR: Thank you; Table 1 caption now appears above the table.

RC: *Caption of Figure 1: Can the authors comment why the lambda differs for 'no feedback' and 'with feedback'?*

AR: We now explain in the Figure 1 caption:

> λ is set using (7) with the mean protein level set to 100. Therefore, $\lambda = 10$ (no feedback), $\lambda = 4.76$ (with feedback).

RC: *Figure 1B right: Can the authors explain why at t=0 the trajectories do not have the same protein level? Is there any pre-simulation done before t=0 that could explain why the green trajectories already start with a much higher level of noise than the blue trajectories?*

AR: As the reviewer intuits, we performed simulations before $t = 0$ so that the selected trajectories start close to steady state. Now, we added in the caption:

> These trajectories are for different single cells with initial values selected from previous simulations such that they start from steady state conditions.

RC: *Line 134: I believe z is not defined.*

AR: Now we explain explicitly that it is the argument of the gamma function, which is set to $= \lambda/\gamma$.

> $z = \lambda/\gamma$ is it argument.

RC: *Line 149: Consider calling the figures in the same order as they are shown. Figure 2B was not yet called in the text.*

AR: Now figures are called in order they are mentioned in the text. We have added the following:

> Figure 2B shows that under weak feedback, there are minimal differences between single-cell and population perspectives, but significant divergence emerges under strong feedback, although the mean concentration remains fixed as per (7).

RC: *x+ is not in Table 1.*

AR: We added this variable to Table 1.

RC: *Consider using the title of each result section to convey the conclusion of that section.*

AR: We thank the reviewer for the suggestion. Now, we have included more subsection titles explaining each main topic.

RC: *Line 318: If cell division is explicitly modeled it is not clear to me why there is a need for a constant dilution rate in the model.*

AR: Our model describes the protein concentration. Other models such as the one proposed by Phillip Thomas describe the number of the proteins. In our burst-dilution model, between consecutive bursts, the cell grows and the concentration decreases because the protein number is fixed. In models that consider cell size homeostasis, the growth/dilution rate is proportional to the division rate. During a division, although cell numbers are halved, cell size is also halved, so that protein concentration is approximately constant, with stochastic variations due to a not perfectly even partitioning of molecules between the descendant cells. This means that, as considered in the first part of the article, division does not change appreciably the protein concentration levels.

To better explain the relationship between dilution and division, we added this explanation in the text:

> The growth rate also defines the rate at which cell division occurs. In our model, division is defined as a point process with a propensity given by (2) with the sole effect, at the population level, of generating a new cell with the same protein level of the parent cell. Therefore, in the single-cell approach, division has no effect on protein concentration.

RC: *Figure 4B caption, squares are red not brown.*

AR: We agree with the reviewer's point. This color is more similar to the "sister" color in Figure 3. We modified the colors such that the color is dark brown.

RC: *Line 25 and 'Author summary': The authors refer several times that single-cell lineage perspective in the commonly used approach. Given the current high throughput techniques available (e.g. flow cytometry, sequencing, etc..), I believe such statement is not accurate nowadays. Can the authors further support this statement or instead use their conclusions to further support the continuation of population wide studies.*

AR: We agree with the reviewer that this claim is debatable and purely subjective. We modified the article to avoid this kind of judgment.

2. Reviewer #2

2.1. Major Comments

RC: *I was not able to understand a point that is fundamental to the claims in the paper: In the single cell model divisions are not represented explicitly. If I understand correctly this is because the concentration is continuous across divisions, and thus divisions have not impact on it. On the other hand, in the population model divisions need to be represented, and they do seem to play an essential role. Therefore, I do not think that we can view the single cell model as a single lineage in the population model. This is in contrast to the work of Philipp Thomas, for example, which does contrast the statistics across a lineage and across a population.*

AR: To address the reviewer's comment, we have studied the Phillip Thomas work, specifically *Thomas, P. (2019): Intrinsic and extrinsic noise of gene expression in lineage trees, Scientific reports, 9(1), 474.* We agree with the reviewer that cell division plays a crucial role in the single-cell lineage (known as *isolated lineage* in Thomas' nomenclature). During each division event, the number of proteins is partitioned.

Unlike Thomas' framework, our approach is concentration-based. During division, if the number of molecules is high enough, partitioning effects can be neglected as the noise component coming from partitioning decays as (number of molecules)$^{-1}$. In this scenario, which is considered in the first part of our approach, division does not significantly alter protein concentration. Both descendant cells, each half the size of the mother cell, inherit half of the molecules, thus maintaining constant concentration during division.

The main difference between our approach and Thomas' lies in the way protein is modeled. However, in terms of population pooling, we believe our population approach is equivalent to Thomas' population approach with unknown age. Similarly, our single-cell perspective corresponds to the single-cell approach with unknown age in Thomas' work.

RC: *This also population distribution exists even when the lineage distribution does not. I do not see how it would be possible that across all lineages the protein levels diverge, in such a way that the population distribution remains stable. I assume something like this is possible, but - if this is what is really claimed - should be explained. In short, the relation between the two models should be clarified a bit more, and the relation to the work of lineage vs population statistics (such ss that of Thomas), should be clarified.*

AR: We agree with the reviewer that the mathematical results are counter-intuitive. To illustrate how the population distribution exists while the single-cell one diverges, we performed individual-based simulations using parameters in which the protein distribution at single cell level diverges but remains bounded at population perspective. Results of those simulations are found in Figure R4.

We include in the main text the following paragraph:

> A more visual approach to the way protein distribution may exist from a population perspective and not at the single cell level can be found in Figure S2. It is possible to observe that in a set of parameters in which the protein distribution exists for the population, the mean protein diverges over time at the single-cell level. This divergence is counterbalanced by increased proliferation of cells with a low protein concentration. This effect maintains the protein distribution at population perspective under steady conditions.

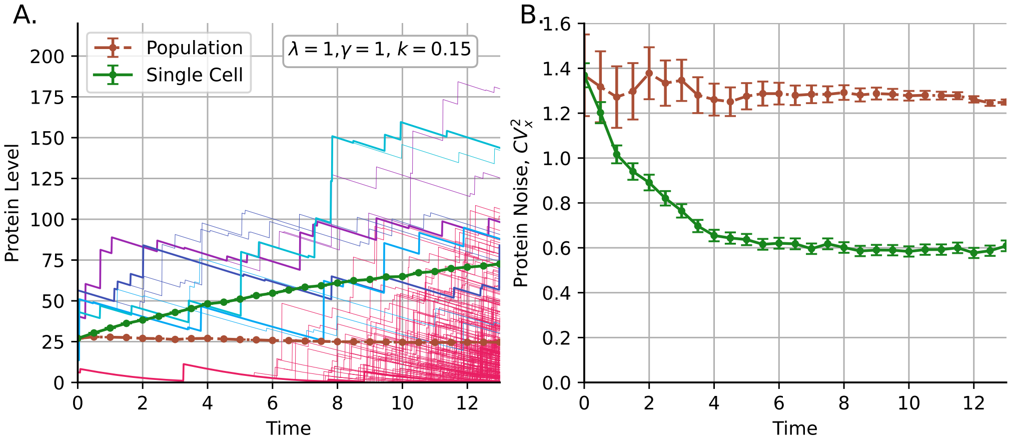

Figure R4: **Dynamics of Protein moments in a context when single-cell protein distribution does not exist but population distribution exists. A.** Examples of protein trajectories. Each color in the background represents cells of the same colony. Green represents the statistics for a single cell approach while brown represent the population perspective. Error bars represents the 95% confidence interval using bootstrapping methods. **B.** Protein noise over time. Statistics were estimated as follows: We simulated 500 colonies with an exponentially growing mean number of individuals. For single cell, 10 individuals were selected randomly with replacement from each colony. For population, the statistics were calculated using all individuals of all colony replicas. The average number of individuals per colony was of 180 after 13 units of time. We performed the simulations using a set of parameters in which single-cell protein distribution does not exist but population distribution exists ($\lambda = 1$, $\gamma = 1$, $k = 0.15$).

2.2. Minor Comments

RC: *In the intro and discussion, it is a little unclear that the authors are talking about lineages. Unless reading carefully, it may see like the discussion is about a single division cycle.*

AR: The reviewer's appreciation is correct. We had referred implicitly Phillip Thomas' work. However, since it is a common reference in the field, we agree that an explicit statement about his contribution is needed. We now add this text:

> This framework (single cell) is also known as *single lineage* since it randomly tracks one of the branches of a lineage tree.

RC: *I also have some questions about the notation: Why are angular brackets used along with a bart to denote the average (eg line 124 and equations below).*

AR: The Chapman-Kolmogorov equation (S.1.1) is, in principle, a time-dependent equation. Therefore, the protein distribution and its moments $\langle x^n \rangle$ are also time-dependent. We use the bar notation to denote the limit of this distribution as $t \to \infty$. The reason for this notation is not clear in the main text, but discriminating between the steady-state moments and the moments at a particular time was necessary in the supplementary appendices.

RC: *A brief discussion of what happens in the limit where only one protein is produced at a time would be interesting (ie what would be missed if bursts were ignored).*

AR: The limit of non-bursty gene expression corresponds to the first part of the approach in which protein production is continuous and deterministic. In that limit, partitioning noise is the only source of noise. The limit of single-molecule production can be achieved when the burst has mean $\langle b \rangle = 1$ and is noiseless $\langle b^2 \rangle = \langle b \rangle^2 = 1$. Since this production occurs at random time, it also contributes to the intrinsic noise. As explained in supplementary equation (S.5.11), the component of noise with a general burst satisfies: $\frac{\gamma}{\lambda}\left(\frac{\langle b^2 \rangle}{2\langle b \rangle^2}\right)$. When the burst is deterministic with size $\langle b \rangle = 1$, the protein noise has half the noise compared to an exponentially distributed burst with the same mean for which $\langle b^2 \rangle = 2$.

RC: *Figure 1a, top: What do the arrows pointing up mean?*

AR: Since a similar inquiry was pointed out by another reviewer, we decide to delete those arrows. The initial purpose for them was to connect the diagram of color tones along the lineage (Figure 1a bottom) with the 2 dimensional plot of protein level over time (Figure 1a top).

RC: *The manuscript could use some editing for language. There are no glaring grammatical errors, but there are many odd phrasings. Improving the presentation would make for an easier reading experience. For example:*

- single-cell studies have interestingly revealed that the second scenario is when - Line 319: 'corresponding', not 'corrupting'.

AR: We thank for the reviewer's suggestions, we edited the article again with a style corrector.

3. Reviewer #3

RC: *1a. One of the main conclusions of this study is that population-wide expression noise is non-negligible when the focal protein affects the cell proliferative capacity, with the assumption that the focal protein is long-lived. The molecular mechanisms of cell division in both prokaryotes and eukaryotes are well understood, and the molecular players (proteins - cyclins and others) are well described. How many of these proteins are long-lived vs. short-lived and actively degraded? The cell cycle necessitates the degradation of at least some of them, for example cyclin D1 has a very short half-life. Can you list examples of long-lived cell cycle proteins (if there are any), as the conclusions of this study are most relevant for these real examples? There are other modulators of cell proliferation which do not control the cell cycle directly, proteins that affect the cell size or growth factors, and would fit the protein of interest criteria. Listing some examples of genes for which the conclusions of the study stand in the discussion would elevate the relevance of the conclusions and provide real-life examples of where the population perspective is not to be ignored when studying expression noise.*

AR: The example pointed out by the reviewer is relevant. However, we believe that division timing regulators are not appropriate examples of proteins that can be explained by our formalism. Among the arguments is that these molecules are not in steady state and are cell-cycle-dependent. This explicit dependence on the cell cycle may require a different approach. For example, this case was studied in *Soltani M, Singh A. (2016): Effects of cell-cycle-dependent expression on random fluctuations in protein levels. R Soc Open Sci 3(12):160578* (citation [62] in the main text). Thus, the framework formulated in this article, if adapted, can be used to explore single-cell and population-level perspectives for such cell-cycle genes in future work.

However, we specify other possible molecules and clarify the issues with the division regulators in the following paragraph in the main text:

> Our approach can also be applied to more direct examples, such as proteins with significant toxicity levels that inhibit cell growth. This scenario is particularly relevant in the case of synthetic constructs where the gene product is non-functional to the cell. Other proteins that affect cell proliferation, such as division timing regulators, may require a more complex cell cycle-dependent modeling.

RC: *1b. Also, the definition of a long-lived protein is relative to the cell-doubling time, i.e. a protein is long-lived if its half-life is significantly longer than the cell-doubling time. Since prokaryotes usually have short cell-doubling times, would more proteins be classified as long-lived in prokaryotes? That would mean that the conclusions apply more frequently to prokaryotic than eukaryotic cells, something the readers would be interested in.*

AR: We agree with the reviewer. To clarify this observation, we added in the text:

> Since prokaryotes usually have short cell-doubling times, the kind of long-lived proteins that can be described by our model can be found mostly in this kind of microorganisms.

RC: *1c. Could you add a short comment on what would happen if the assumption of no degradation was relaxed? Do you expect it to change the results?*

AR: Thank you for your question. If we relax the assumption of no degradation and instead assume that the protein is short-lived, it will naturally degrade at a constant rate $\gamma_{\text{deg}} > 0$ (here and below, we add "deg" to the article notation to distinguish the new model with non-zero degradation rate). This affects only the deterministic

decay of the protein between consecutive bursts, now described by $\dot{x}(t) = -x\frac{\gamma}{1+kx} - \gamma_{\text{deg}}x$. Following the same solution approach, we obtain that in a single-cell perspective, the steady-state distribution is following:

$$p_{SC}^{\text{deg}}(x) = \frac{\psi(1+kx)(\psi x)^{\frac{\lambda}{\gamma+\gamma_{\text{deg}}}-1}(\psi x+1)^{\frac{\lambda\gamma}{\gamma_{\text{deg}}(\gamma+\gamma_{\text{deg}})}-1}e^{-x/\beta}}{U\left(\frac{\lambda\psi}{\gamma_{\text{deg}}k}, \frac{\lambda}{\gamma_{\text{deg}}}, \frac{1}{\psi\beta}\right) + \frac{\lambda}{\gamma_{\text{deg}}}U\left(\frac{\lambda\psi}{\gamma_{\text{deg}}k}+1, \frac{\lambda}{\gamma_{\text{deg}}}+1, \frac{1}{\psi\beta}\right)}, \tag{R4}$$

where $\psi = \gamma_{\text{deg}}k/(\gamma + \gamma_{\text{deg}})$ is a positive constant and $U(\cdot,\cdot;\cdot)$ is the Tricomi function (the confluent hypergeometric function of the second kind).

The protein distribution (R4) yields the n-th steady-state raw moment of the protein concentration:

$$\overline{\langle x^n\rangle}_{SC}^{\text{deg}} = \frac{\left(\frac{\lambda\psi}{\gamma_{\text{deg}}k}\right)_n}{\psi^n} \cdot \frac{U\left(\frac{\lambda\psi}{\gamma_{\text{deg}}k}+n, \frac{\lambda}{\gamma_{\text{deg}}}+n, \frac{1}{\psi\beta}\right) + \left(\frac{\lambda}{\gamma_{\text{deg}}}+\frac{nk}{\psi}\right)U\left(\frac{\lambda\psi}{\gamma_{\text{deg}}k}+n+1, \frac{\lambda}{\gamma_{\text{deg}}}+n+1, \frac{1}{\psi\beta}\right)}{U\left(\frac{\lambda\psi}{\gamma_{\text{deg}}k}, \frac{\lambda}{\gamma_{\text{deg}}}, \frac{1}{\psi\beta}\right) + \frac{\lambda}{\gamma_{\text{deg}}}U\left(\frac{\lambda\psi}{\gamma_{\text{deg}}k}+1, \frac{\lambda}{\gamma_{\text{deg}}}+1, \frac{1}{\psi\beta}\right)}. \tag{R5}$$

Namely, the mean protein concentration in a single cell is (Fig. R5B):

$$\overline{\langle x\rangle}_{SC}^{\text{deg}} = \frac{\lambda}{\gamma_{\text{deg}}k} \cdot \frac{U\left(\frac{\lambda\psi}{\gamma_{\text{deg}}k}+1, \frac{\lambda}{\gamma_{\text{deg}}}+1, \frac{1}{\psi\beta}\right) + \frac{\lambda+\gamma+\gamma_{\text{deg}}}{\gamma_{\text{deg}}}U\left(\frac{\lambda\psi}{\gamma_{\text{deg}}k}+2, \frac{\lambda}{\gamma_{\text{deg}}}+2, \frac{1}{\psi\beta}\right)}{U\left(\frac{\lambda\psi}{\gamma_{\text{deg}}k}, \frac{\lambda}{\gamma_{\text{deg}}}, \frac{1}{\psi\beta}\right) + \frac{\lambda}{\gamma_{\text{deg}}}U\left(\frac{\lambda\psi}{\gamma_{\text{deg}}k}+1, \frac{\lambda}{\gamma_{\text{deg}}}+1, \frac{1}{\psi\beta}\right)}. \tag{R6}$$

The analytical results (R4) and (R6) were validated through simulations of the corresponding model.

Including natural degradation in the population model significantly increases complexity. Specifically, the ODE for the probability distribution $p_{POP}^{\text{deg}}(x)$ becomes a confluent form of the Heun equation. Solving this equation remains an area for future research; hence, we relied on simulations to demonstrate the effects of γ_{deg} on the population distribution.

Compared to long-lived proteins, the steady-state distribution for short-lived proteins exists for any set of positive parameters $\alpha, \beta, k, \gamma, \gamma_{\text{deg}}$. This result is expected, as the existence conditions were initially caused by the accumulation of protein in a cell due to the cell-growth-dependent dilution rate. Adding an independent source of protein concentration decrease – natural degradation – resolves this issue. The simulations showed that $p_{POP}^{\text{deg}}(x)$ also exists for any set of positive parameters $\alpha, \beta, k, \gamma, \gamma_{\text{deg}}$, following the same reasoning as in the single-cell case.

We compare the obtained results with the single-cell and population models of long-lived protein (i.e. case studied in the article), which is shown in Fig. R5A. For correct comparison we hold the mean protein level in all single-cell models fixed. When the dilution rate is the dominant cause of protein decay (i.e., $\gamma > \gamma_{\text{deg}}$), $p_{SC}^{\text{deg}}(x)$ and $p_{POP}^{\text{deg}}(x)$ are close to $p_{SC}(x)$ and $p_{POP}(x)$, respectively (Fig. R5A). As degradation becomes dominant ($\gamma \leq \gamma_{\text{deg}}$), the feedback effect becomes negligible, leading to almost unregulated protein decay. Thus both distributions $p_{SC}^{\text{deg}}(x)$ and $p_{POP}^{\text{deg}}(x)$ become almost identical (Fig. R5C). As γ_{deg} increases, both distributions approach the gamma distribution corresponding to the unregulated case ($p_{\text{unreg}}(x) = \Gamma(\lambda/\gamma_{\text{deg}}, \beta)$).

RC: *2. One of the main methods of studying stochasticity of gene expression is through single-cell measurements of protein level or sequencing of isogenic cell populations, e.g. single-cell RNA-seq, both of which would correspond to a population perspective in the text. However, results in terms of expression level distribution and variance from scRNA-seq data are often used to make claims about the expression dynamics within single cells, with the assumption that they can be equated. This study is in a unique position to study both and*

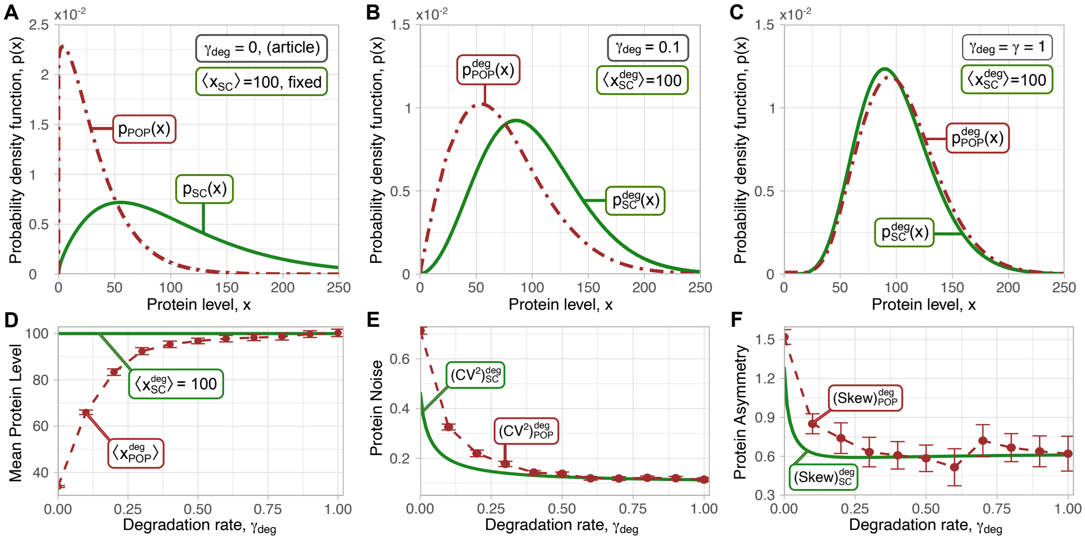

Figure R5: **When the gene product is unstable, the distributions for single cell and population converge** Comparison of protein distributions in single-cell (green solid lines) and population (red dot-dashed lines) perspectives as: (**A**) the protein is stable and the degradation rate is equal to zero (case studied in the article); **(B)** the degradation rate is slower then the maximum dilution rate ($\gamma_{\text{deg}} = \gamma/10 = 0.1$); **(C)** the degradation rate is equal to the maximum dilution rate ($\gamma_{\text{deg}} = \gamma = 1$). **(D)** Mean protein concentration, **(E)** Protein noise quantified by the squared coefficient of variation and **(E)** The protein asymmetry quantified by the skewness for both frameworks: single cell (green) and population (brown error bars). In all cases, the burst frequency λ is set such that $\overline{\langle x \rangle}_{SC} = \overline{\langle x \rangle}_{SC}^{\text{deg}} = 100$ using (R6). Other parameters: $k = 0.05$, $\beta = 10$, $\gamma = 1$. The moments for the population perspective where calculated from 2000 simulated colonies. The error bars represent 95% confidence intervals using bootstrapping. The distributions $p_{SC}^{\text{deg}}(x)$ are given by (R4) and $p_{POP}^{\text{deg}}(x)$ is obtained using boundary-corrected density estimation from simulations.

compare them, and I think it would be impactful to point out in the discussion when the two perspectives can be equated. This is even mentioned in the early papers studying noise (e.g. Friedman et al. 2006, cited in the manuscript) – that we are not sure if the ergodic principle from statistical physics holds for this system, in other words, whether the statistical distribution of the ensemble, the population of cells, is identical to the distribution of states over time in one process, within one cell. The scenarios in which the two perspectives yield different distribution is important, and correctly highlighted in the manuscript, but the opposite, that in many cases the distributions do not differ at all, is also insightful. Particularly since the single-cell perspective approach, to observe gene expression levels in real time, is a much more demanding experimental setup than the population approach. If one can claim that the expression distribution of one gene out of thousands of genes analyzed in an RNA-seq experiment are identical to the expression distribution of this gene over time within one cell, there is no need to do tedious massive scale GFP tagging to observe the dynamics of each individual gene in the genome.

AR: We agree with the reviewer's point. From the single-cell perspective, it is clear that the ergodic principle applies as long as the measurements over the time are sampled during a period of time longer than the autocorrelation typical decay time. In the population perspective, we observe that protein density consists of an exponentially growing function times a distribution that reaches a fixed value at the limit $t \to \infty$. Now, in the discussion, we added the following paragraph:

> Although we highlighted the differences between single-cell and population approaches, it is also important to mention the scenarios where single-cell statistics are similar to population statistics. First, we found:
>
> - When the protein half-life is short relative to the cell cycle (Fig. S4).
>
> When the protein half-life is long, we observe additional regimes:
>
> - When partitioning noise is much more smaller than intrinsic noise, and the protein level does not affect cell growth or proliferation.
> - When the burst frequency is proportional to the growth/dilution rate. Within the explored parameter regime, the differences between both approaches decrease significantly (Fig. S3).
>
> These equivalent scenarios are relevant for experimental design. By measuring protein statistics in one scenario, it is possible to claim that the protein statistics in the other scenario will be equivalent. Our work provides a framework for estimating the degree of similarity between these population perspectives.

RC: *3. I think reordering two paragraphs in the introduction would yield a better reading flow. Paragraph starting at line 33 describes the first scenario in which the population and single-cell distribution differ, then paragraph on line 44 describes the mathematical methods used in both perspectives, then paragraph on line 51 goes back to the second scenario. It would be better to describe the first scenario, then second, then finish with the mathematical description of both (i.e. swap paragraph on line 51 and 44).*

AR: Thank you for the suggestion. However, as each scenario utilizes a different mathematical framework to model and analyze the respective phenomena, we believe the current flow is more appropriate. To address potential confusion, we have clarified this distinction in the text.

RC: *4. Line 82: why is "one-dimensional" used to describe the model of gene expression? It would be simpler to just write the telegraph model of gene expression, which is how it is commonly referred to.*

AR: A complete approach to gene expression considers at least two dimensions: the level of RNA and the level of protein. Here, the noise transmitted from the RNA is included in the effective parameter burst size. In this way, the gene expression system can be simplified to a one-dimensional system. The single dimension corresponds to the protein level.

RC: *5. Line 117: Text says that the case of no feedback (k = 0) is explored, while in the figure the data for weak feedback is shown (k=0.005).*

AR: We think there was a misunderstanding. In the mentioned line, we refer to Figure 1B and not Figure 2B which probably the reviewer refers to. In Figure 1B top, we show trajectories of cell protein over time with no feedback.
