# Supplementary material for: Stochastic gene expression in proliferating cells: Differing noise intensity in single-cell and population perspectives
Source: PLoS Comput Biol. 2025 Jun 10;21(6):e1013014. doi: 10.1371/journal.pcbi.1013014 (PMC12151482; doi:10.1371/journal.pcbi.1013014)
Supplement: Attachment — Submitted filename: PLoS_Author_response_to_reviewers__Copy2025_.pdf [file pcbi.1013014.s006.pdf]

Authors’ Response to Reviews on Manuscript (PCOMPBIOL-D-24-01119) - [EMID:a091fed37246d806]

Stochastic Gene Expression in Proliferating Cells: Differing Noise Intensity in Single-Cell and Population Perspectives.

by the Manuscript’s Authors
PLoS Computational Biology

***RC:** Reviewers’ Comment*, **AR**: Authors’ Response, ☐ Manuscript Text

The authors thank the reviewers for their feedback. Their suggestions greatly improved the quality of the article. In this response we address the suggestions of reviewer 1.

1. Reviewer #1

1.1. Major Comments

RC: *- Could the authors verify if the text referred to ‘added to the main article’ in the response letter is indeed included in the main manuscript. For example, I could not find the text below in response to Question 1. ‘We also studied how single cell and population statistics differ considering that transcription rate is not constant but rather depends on cell growth rate. In the context in which the transcription rate is proportional to the growth rate, we observe that the feedback strength induces smaller differences in the protein moments between the mean and the protein perspectives. The mean at population level is round 10% less than the mean for single cell while the noise is essentially the same between both perspectives.’*

AR: We apologize to the reviewer. The mentioned text appears in the third paragraph of the subsection "Complementary formulations for protein statistics". In pas response both versions differed slightly since the final text was shortened in comparison with the version written in the rebuttal letter. We added the details we missed in the previous version and now the paragraph reads:

> As a second scenario, we studied how single cell and population statistics differ if the transcription rate is not constant but depends on the cell growth rate ($\lambda(x) \propto \gamma(x)$). In this context, we observe that the feedback strength induces smaller differences in the protein means between the single-cell and the population perspectives. The mean at population level is round 10% less than the mean for single cell while the noise is essentially the same between both perspectives. Details of this model are provided in Appendix S4.

RC: *– Fig. R1: Considering adding y-axis label in C and D.*

AR: We added the suggested labels. The mentioned figure now corresponds to Figure S3 in supplemental Information, the new plot is presented in Fig. R1:

RC: *– Consider changing colors in Fig. 3 and 4. Figure 4B shows population as brown squares. Fig 3C-D show it as red squares. Moreover, the brown color is also not very distinguishable from the red color used to identify sister cell paths in 3B, 4A and C. As such, it gives the idea that the data is extracted from the sister cells trajectories. I suggest changing the color of population metrics (3C, 3D, 4B) and population distribution plots*

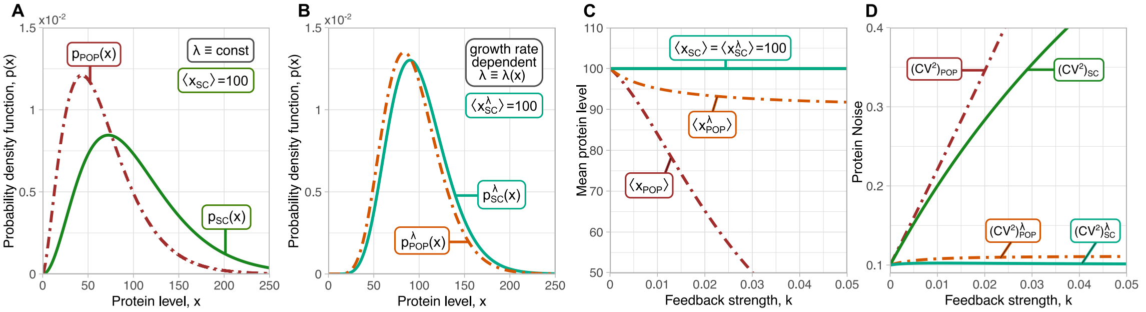

Figure R1: **Difference between single cell and population perspectives is less prominent when the transcription rate is proportional to the cell growth rate**. Comparison of protein distributions in single-cell and population perspectives as: (**A**) the burst frequency is independent from protein concentration, $\lambda_{\max}$ is chosen so that $\overline{\langle x\rangle}_{SC} = 100$ (case studied in our article); (**B**) the burst frequency is protein-dependent, $\lambda_{\max}$ is again chosen so that $\overline{\langle x\rangle}^{\lambda}_{SC} = 100$ as per (S5.3). (**C**) Mean protein concentration and (**D**) protein noise in both models. For all plots $k = 0.02$, $\beta = 10$, $\gamma = 1$.

(4A, 4C, 3B, right-side distributions) to the same light brown color as 'Other cells' or a new distinguishable color.

The authors thank the reviewer for pointing out the color discrepancy. Now, we update the figures showing a darker brown when plotting the statistics at population perspective. updated plots are shown in Fig. R2 and Fig. R3. We use a color different from "other cells" since the statistics include all cells (progenitor, sisters, and other cells):

RC: *Line 86: 'The first method involves taking the time-averaged statistics of the stochastic process over a long trajectory (as shown in Fig. 1A). The second method involves estimating the statistics at a given time across different replicas (as illustrated in Fig. 1B)' I am slightly confused, in Fig.1A the illustration shows that the population distribution is drawn at a given point (last one). Meanwhile, Figure 1B shows the distributions being drawn from the statistics of single cells over time. As such, I believe 1A and 1B should be switched in the sentence above.*

AR: We thank the reviewer for the inquiry. We modified the text as follows:

> The first method involves taking the time-averaged statistics of the stochastic process over a long trajectory (as shown in Fig. 1A top in which the statistics are obtained from a single trajectory). The second method involves estimating the statistics at a given time across different replicas (as illustrated in Fig.1B in which the statistics are estimated from multiple independent trajectories at the last point).

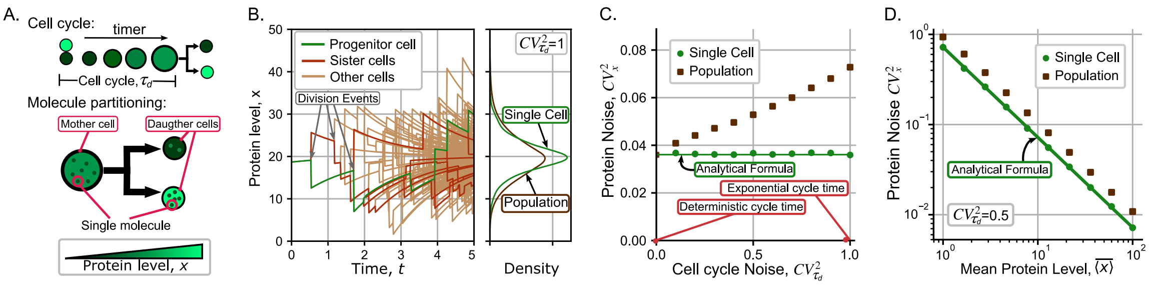

Figure R2: **Noisy cell-cycle durations amplify protein noise differences between single-cell and population perspectives.** (**A**) *(top:)* The cell-cycle duration τ_d is a random variable following an arbitrary distribution. Within the cell cycle, the protein concentration evolves deterministically as per the ordinary differential equation (15)). *(bottom:)* During mitosis, protein molecules are randomly segregated among daughters resulting in differences in the inherited concentration. Different shades of green represent different levels of protein concentration. (**B**) *(left:)* Trajectories of protein concentration in an expanding cell colony, where jumps represent randomness in protein partitioning among daughters during cell division. The green line: a single-cell trajectory is generated by randomly choosing one of the two daughter cells (red lines) after each division event. The light brown lines represent other descendant cells. The cell-cycle times are assumed to be exponentially distributed in this simulation. Single cell statistics are estimated over a 5000 independent individuals; population statistics are estimated using all cells of 2000 colonies (including sisters, progenitor and other cells). Statistics were calculated after 6 generations. *(right:)* The steady-state probability density functions of the protein concentration in single-cell and population perspectives. (**C**) Effect of noise in the cell cycle time as quantified by its squared coefficient of variation ($CV^2_{\tau_d}$) on the noise in the protein concentration (CV^2_x). The solid line is the analytically predicted noise in the single-cell perspective as given by (18), and the dots represent noise levels computed from simulations. Mean concentrations in both models are identical ($\overline{\langle x \rangle}_{SC} = \overline{\langle x \rangle}_{Pop} = 20$). (**D**) A logarithmic scale representation of the steady-state protein noise level as a function of the mean protein level, highlighting variability differences between single-cell and population perspectives. Parameters used for the plot are $\gamma = \ln 2$, $\langle \tau_d \rangle = 1$, $\varepsilon = 1$, $k\beta = 20\gamma$.

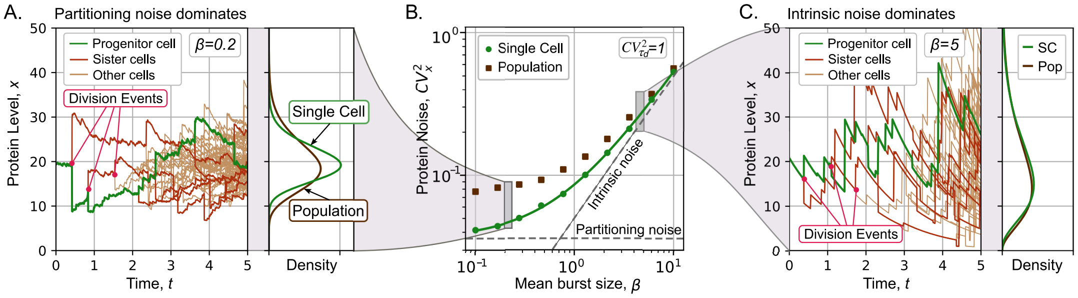

Figure R3: **Increasing randomness in molecular segregation between daughters enhances protein noise differences between single-cell and population perspectives**. (**A**) Sample trajectories of protein concentration in an expanding cell colony when expression variability is dominated by partitioning noise ($\beta = 0.2$ and $\varepsilon = 1$ in (20)). (**B**) Comparison of the steady-state protein concentration noise (CV^2_x) from single-cell (green circles) and population perspectives (brown squares) calculated from simulations of the agent-based model. The solid line represents the analytically-predicted noise level (20). (**C**) Sample concentration trajectories for the high intrinsic noise scenario ($\beta = 5$ and $\varepsilon = 1$ in (20). Other parameters are taken as $\gamma = \ln 2$, $\langle x \rangle = 20$, $\lambda = \gamma \langle x \rangle / \beta$, $\varepsilon = 1$). 2000 colonies where simulated for population perspective, 5000 individuals where simulated for single-cell perspective.
