# Supplementary material for: Stochastic gene expression in proliferating cells: Differing noise intensity in single-cell and population perspectives
Source: PLoS Comput Biol. 2025 Jun 10;21(6):e1013014. doi: 10.1371/journal.pcbi.1013014 (PMC12151482; doi:10.1371/journal.pcbi.1013014)
Supplement: Attachment — Submitted filename: Review.docx [file pcbi.1013014.s002.docx]

***Review of ‘Stochastic Gene Expression in Proliferating Cells: Differing Noise Intensity in Single-Cell and Population Perspectives’***

The study focuses on investigating protein noise levels (random fluctuations in the level of a given protein) from two different perspectives: 1) expression in a single cell lineage over time and, 2) expression between cells in a population, at a given time point (as illustrated in Figure 1). For this, the authors developed a stochastic model with molecular bursting of gene products and analytically predict the steady-state distributions of protein concentration in both perspectives. Specifically, the authors investigate two scenarios where both perspectives lead to different noise levels. The first scenario corresponds to high protein concentrations inhibiting cell division. Using analytical derivations for the statistical properties of the steady-state distributions, the authors show that, for weak feedback strength in growth dynamics, the distributions of single-cell and population perspective do not differ. Whereas, for strong feedback, the population perspective shows lower protein levels and higher noise and skewness (Figure 2). The second scenario regards the noise inherent to the partitioning of cellular contents, during cell division. The authors show that randomness in cell cycle time has no impact in the protein noise level for the single cell perspective. Conversely, for the population perspective, the protein noise level increases with increasing randomness in cell-cycle times (Figure 3). Finally, the authors also show that the steady-state protein distributions of both perspectives only differ for the case in which partitioning noise dominates the intrinsic noise (i.e., burst size) (Figure 4). Overall, the results show that single cell lineages can underestimate noise levels. Although, I find the main conclusion not novel, I believe the work systematically shows the importance of doing population studies to evaluate noise levels, specially for proteins controlling growth dynamics. Further, I believe the analytical derivations reported are of high relevance to predict the adaptation capabilities of cells to different environments. Nevertheless, I find that further efforts can be made to validate the predictions with previously reported empirical data. My comments are categorized below into major and minor comments/questions/suggestions.

**Major comments:**

1) The current model does not account for the fact that gene expression rates are cell growth rate dependent. Are the results shown in Figure 2 affected if this dependence is included in the model?

2) Figure 2B and C – I believe the results for the statistical properties presented are based on the analytical predictions from equations (4) (for the population) and from equations (6a-6c) (for the single cell). Can the authors show how well the model simulations results fit these predictions?

3) Line 78: *‘Protein statistics are determined based on a single lineage path over time’…in the population approach, both daughter cells are tracked, and statistics…are estimated at a fixed time point’*. Given this description, I am not sure what kind of distributions are illustrated in Figure 1B. Are they single cell or population distributions?

4) Section: *‘Coupling deterministic expression with partitioning error’*. Can the authors clarify why only at this stage the authors decided to change the cell cycle assumption. Do the results shown in Figure 2 are robust to the relaxation of the cell cycle assumption?

5) Throughout the manuscript the authors claim to have studied/identified two scenarios for which the protein noise of the two perspectives differs. However, an extensive part of the manuscript is dedicated to the effects of randomness in cell-cycle times while keeping *epson* (i.e. randomness level of partitioning) constant. Could the randomness in cell-cycle times be identified as a third scenario for which protein noise differs in both perspectives (as seen in Figure 3B) ?

6) Can the authors clarify if the distribution for the population perspective in Figure 3B, 4A and 4C is taken across all descendant cells (red+brown) or solely sister cells (red)? Due to the color scheme, I assume it is the latter, however, Figure 1B bottom considers both ‘sister’ and ‘other cells’ to explain the population perspective.

7) Line 333 reads *‘at a fixed time point’* (as well as in many other parts of the manuscript, e.g. line 80). However, according to the red trajectories and red distributions in Figure 3B, 4A and 4C, I assumed that the descendant cells were sampled at several fixed time points to draw the population level distribution and not solely at last time point, as depicted in Figure 1B bottom. Could the authors clarify which approach was used and perhaps update Figure 1B accordingly?

8) Differences between population and single-cell distributions are found only when protein levels have feedback in the growth dynamics. Can the authors discuss how relevant is it to study this kind of proteins? Do they exist in abundance? Or is this feedback mostly due a certain protein reaching toxicity levels for the cell, thus halting cell division?

9) Works have shown that a good strategy is to binomially partition the cell contents (e.g. doi:10.1371/journal.pone.0079345). Can the authors explain why binomial partition was not considered?

10) Line 78: *‘Protein statistics are determined based on a single lineage path over time’*. Should it read instead: ‘ Protein statistics are determined based on multiple single lineage paths over time’. Since as described in Figure 4 caption, 5000 cells were studied.

11) Line 314 and 315: Suggests that partitioning noise is not a form of intrinsic noise. However, line 382 calls it *‘another source of intrinsic noise’*. Can the authors considering clarifying what they consider to be intrinsic noise?

12) As the authors discuss, it would be of great interest to validate these predictions with empirical data. For example, one could probably make use of the data (time-lapse protein expression levels for single lineage and descendant cells) shown in Figure 2C of (DOI: 10.1126/science.1188308). Further, the same type of data at the mRNA level can also be found in (DOI:10.1371/journal.pcbi.1005174).

**Minor comments:**

1) Line 64: Should it read ‘Single cell and population *analysis …’ ?*

2) Fig 1A: What is the meaning of the four upward grey arrows? Also, are the vertical lines in the single-cell protein level illustration denoting cell division events? Are they supposed to match the division events of the root of the three?

3) Table 1: Table captions should be on top of the table

4) Caption of Figure 1: Can the authors comment why the *lambda* differs for ‘no feedback’ and ‘with feedback’ ?

5) Figure 1B right: Can the authors explain why at t=0 the trajectories do not have the same protein level? Is there any pre-simulation done before t=0 that could explain why the green trajectories already start with a much higher level of noise than the blue trajectories?

6) Line 134: I believe *z* is not defined.

7) Line 149: Consider calling the figures in the same order as they are shown. Figure 2B was not yet called in the text.

8) *x+* is not in Table 1.

9) Consider using the title of each result section to convey the conclusion of that section.

10) Line 318: If cell division is explicitly modeled it is not clear to me why there is a need for a constant dilution rate in the model.

11) Figure 4B caption, squares are red not brown.

12) Line 25 and ‘Author summary’: The authors refer several times that single-cell *lineage* perspective in the commonly used approach. Given the current high throughput techniques available (e.g. flow cytometry, sequencing, etc..), I believe such statement is not accurate nowadays. Can the authors further support this statement or instead use their conclusions to further support the continuation of population wide studies.
