# Supplementary material for: Stochastic gene expression in proliferating cells: Differing noise intensity in single-cell and population perspectives
Source: PLoS Comput Biol. 2025 Jun 10;21(6):e1013014. doi: 10.1371/journal.pcbi.1013014 (PMC12151482; doi:10.1371/journal.pcbi.1013014)
Supplement: Attachment — Submitted filename: Review_02.docx [file pcbi.1013014.s005.docx]

**Review 02 of ‘Stochastic Gene Expression in Proliferating Cells: Differing Noise Intensity in Single-Cell and Population Perspectives’**

The authors made a significant effort to answer all my comments. The changes introduced addressed all my initial concerns. I am happy to see the manuscript accepted. The following are minor suggestions I believe could be implemented to further improve the manuscript:

**Minor comments:**

01- Could the authors verify if the text referred to ‘added to the main article’ in the response letter is indeed included in the main manuscript. For example, I could not find the text below in response to Question 1.

‘We also studied how single cell and population statistics differ considering that transcription rate is not constant but rather depends on cell growth rate. In the context in which the transcription rate is proportional to the growth rate, we observe that the feedback strength induces smaller differences in the protein moments between the mean and the protein perspectives. The mean at population level is round 10% less than the mean for single cell while the noise is essentially the same between both perspectives.’

02 – Fig. R1: Considering adding y-axis label in C and D.

03 – Consider changing colors in Fig. 3 and 4. Figure 4B shows population as brown squares. Fig 3C-D show it as red squares. Moreover, the brown color is also not very distinguishable from the red color used to identify sister cell paths in 3B, 4A and C. As such, it gives the idea that the data is extracted from the sister cells trajectories. I suggest changing the color of population metrics (3C, 3D, 4B) and population distribution plots (4A, 4C, 3B, right-side distributions) to the same light brown color as ‘Other cells’ or a new distinguishable color.

04 – Line 86: ‘The first method involves taking the time-averaged statistics of the stochastic process over a long trajectory (as shown in Fig. 1A). The second method involves estimating the statistics at a given time across different replicas (as illustrated in Fig. 1B)’

I am slightly confused, in Fig.1A the illustration shows that the population distribution is drawn at a given point (last one). Meanwhile, Figure 1B shows the distributions being drawn from the statistics of single cells over time. As such, I believe 1A and 1B should be switched in the sentence above.
